# High-Performance Photochromic Diarylethene with a Boron–Nitrogen Heterocyclic Ethene Bridge

**DOI:** 10.3390/molecules31071115

**Published:** 2026-03-28

**Authors:** Chen Zhang, Yuping Dai, Yilin Chen, Shaoqiang Dong, Jiaxing Wang

**Affiliations:** 1 School of Materials Science and Engineering, Dongguan University of Technology, Dongguan 523808, China; 2 Institut des Sciences Analytiques et de Physico-Chimie pour l’Environnement et les Matériaux IPREM UMR 5254, Université de Pau et des Pays de l’Adour, 64053 Pau, France; 3 State Key Laboratory of Advanced Materials for Intelligent Sensing and Key Laboratory of Organic Integrated Circuits, Ministry of Education & Tianjin Key Laboratory of Molecular Optoelectronic Sciences, Department of Chemistry, Institute of Molecular Aggregation Science, Tianjin University, No. 92 Weijin Road, Nankai District, Tianjin 300072, China

**Keywords:** photochromism, diarylethene, boron–nitrogen heterocycle, isosterism

## Abstract

Photochromic diarylethene molecules are promising candidates for applications in optical data storage devices. However, many reported diarylethene compounds suffer from inefficiencies due to low photocyclization quantum yields or poor fatigue resistance. To address this issue, we have developed a highly efficient boron–nitrogen heterocycle-bridged diarylethene. The bulky boron–nitrogen heterocyclic ethene bridge blocks interconversion between parallel and *anti*-parallel conformations, yielding two separated rotamers. Evaluation of their photochromic properties demonstrated that the *anti*-parallel conformer exhibits a high photocyclization quantum yield (Φ_o-c_, 89.2%), excellent thermodynamic stability at 298 K and moderate fatigue resistance in hexane. Furthermore, direct comparison with its isosteric carbonaceous analog revealed that incorporating the azaborine moiety into the diarylethene scaffold significantly enhances its photochromic performance. This work presents a strategy that employs azaborine chemistry for the development of potential diarylethene-based photoswitchable materials.

## 1. Introduction

Photochromic dyes, characterized by their distinguishable color changes upon light irradiation, are key components for developing smart materials [1,2,3,4]. Among these, diarylethene (DAE) derivatives are particularly promising for applications in photoswitching [5], data storage [6], and logic gates [7], owing to the perfect reversibility and distinct absorption profiles of their open and closed forms. Pursuing ideal performance—defined by high photoconversion efficiency, robust thermal stability, and exceptional fatigue resistance—has driven extensive research into the synthesis and modification of diverse diarylethene frameworks [8,9,10]. Since Irie developed the first example of a DAE [11], the most common diarylethenes are dithienylethenes, featuring a perfluorocyclopentene as the ethene bridge [12,13,14]. Apart from derivatizing the substituents of the perfluorocyclopentene framework, incorporation of different heterocycles, such as quinoline [15], indole [16], 1,10-phenanthroline [17], *N*-heterocyclic carbene [18], pyridylimidazole [19], β-diketonate [20], and phosphole [21], into the bridging part is one of the most efficient approaches to optimize the photochromic properties. Most of these molecules exhibit a relatively low photocyclization quantum yield and poor stability of the closed form, which limits their further application in photoresponsive materials. Despite this, several heterocycle-bridging diaryethenes demonstrate remarkable photochromic performance, such as benzothiadiazole-fused dithienylethene [22,23]. In particular, the sterically hindered *anti*-parallel benzothiadiazole-fused dibenzothienylethene [24] has a high photocyclization quantum yield of up to 90.6%, as reported by Zhu, Tian and co-workers. The silole- and germole-fused dithienylethenes reported by Yam and co-workers demonstrated a high thermal irreversibility and excellent fatigue resistance [25,26].

In parallel to these developments in classic heterocycles, the emerging field of azaborine chemistry provides a novel alternative for material design due to their less aromatic nature than all-carbon polycyclic aromatic hydrocarbons (PAHs) [27,28]. The replacement of a carbon–carbon unit with an isoelectronic boron–nitrogen unit has been proven as a powerful tool to regulate the photophysical and optical properties of PAHs [29,30]. A large number of reported boron–nitrogen-embedded PAHs have been applied in organic light-emitting diodes (OLEDs) [31], organic field-effect transistors (OFETs) [32] and singlet fission materials [33]. Despite the usefulness of the BN/CC isosterism approach in enriching PAH chemistry, surprisingly, there are no reports of its application in photochromic diarylethene to date. To the best of our knowledge, only a few examples of the use of a tetrahedral boron framework as the bridge part of diarylethene have been reported [34,35]. In this paper, we describe the syntheses of a boron–nitrogen-containing heterocycle-based diarylethene and its carbonaceous analog. This brand-new boron–nitrogen heterocycle ethene bridge endows diarylethene with excellent photochromic performance, including a high photocyclization quantum yield, high thermodynamic stability at ambient conditions and moderate fatigue resistance in hexane. The photophysical and photochromic properties are investigated in detail to evaluate the difference in photochromic behavior between the isosteres. Insights into the electronic structure of the compound and the nature of its excited states have also been provided by computational studies.

## 2. Results and Discussion

**Scheme 1:**
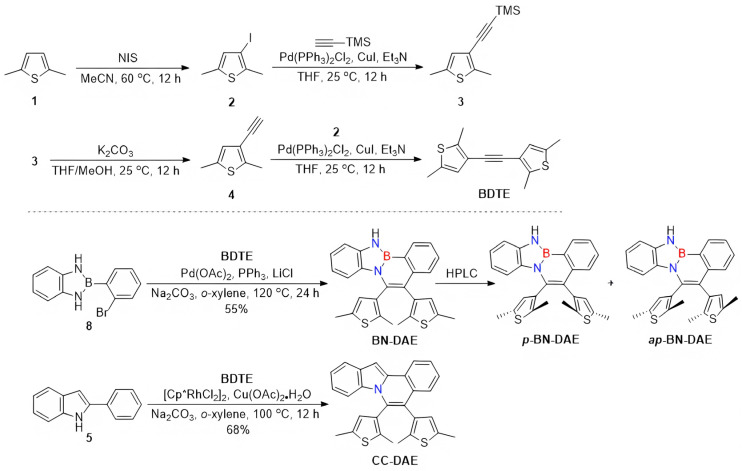
Syntheses of ***p*-BN-DAE, *ap*-BN-DAE** and **CC-DAE**.

The details of the syntheses are shown in Scheme 1. Firstly, the precursor 1,2-bis(2,5-dimethylthiophen-3-yl)ethyne (BDTE) was prepared according to previously reported procedures with a slight modification [25]. Iodination of 2,5-dimethylthiophene, followed by Sonogashira cross-coupling with ethynyltrimethylsilane, yielded compound **3**. Subsequently, deprotection of **3** and Sonogashira cross-coupling with **2** delivered BDTE in good yield. Using reported methodologies, **BN-DAE** and **CC-DAE** were readily prepared via palladium-catalyzed cyclization [36] of **8** with BDTE and rhodium-catalyzed cyclization [37] of 2-phenylindole with BDTE, respectively. The steric interaction between the thiophenes and the heteroarene bridge leads to hindered rotation of the thiophenes. Consequently, both parallel (*p*-) and *anti*-parallel (*ap*-) conformers were observed in the proton NMR spectrum, and we successfully isolated rotamers of **BN-DAE** as ***p*-BN-DAE** and ***ap*-BN-DAE** using preparative high-performance liquid chromatography (HPLC) with a common C18 column. The ratio of ***ap*-BN-DAE** to ***p*-BN-DAE** was 57:43, consistent with previous reports that the *ap*-conformer is the preferred conformation. We found that in both parallel and *anti*-parallel conformers, there is no interconversion in acetonitrile at room temperature for at least 48 h. Due to their poor solubility in acetonitrile, the rotamers of **CC-DAE** could not be separated. The ratio of its two conformations was determined to be 55:45 by analytical HPLC. ***p*-BN-DAE**, ***ap*-BN-DAE** and **CC-DAE** were characterized by ^1^H, ^13^C, and ^11^B (only for boron-containing compounds) NMR spectroscopy and high-resolution mass spectroscopy. Unfortunately, we were not able to obtain a single-crystal structure of **BN-DAE**. Meanwhile, a yellow needle-like crystal of **CC-DAE** was collected by slow evaporation of the solution in CH_2_Cl_2_/MeOH (4:1, *v*/*v*) at 0 °C. X-ray diffraction analysis revealed a disordered arrangement in the crystal structure. This disorder could be explained by the coexistence of molecules with parallel (meso) and *anti*-parallel (racemic) configurations within the same crystal lattice [38].

**Figure 1:**
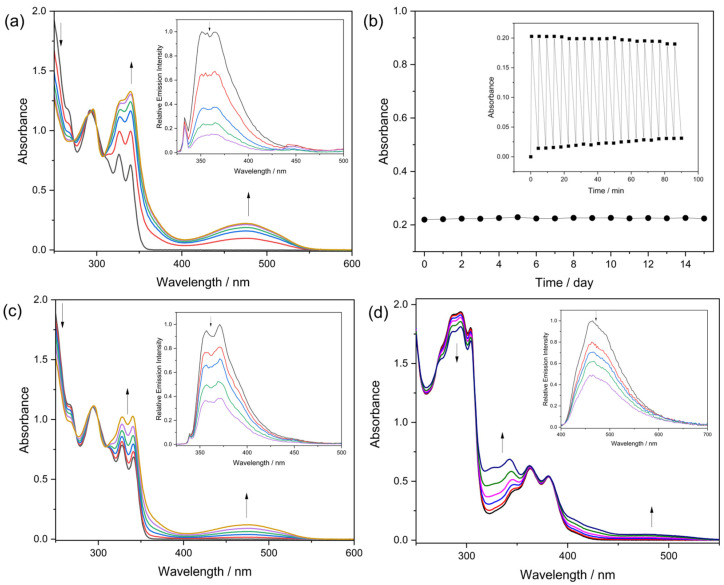
(**a**) Changes in the absorption spectrum of ***ap*-BN-DAE** upon irradiation at 254 nm in hexane (5 × 10^−5^ mol/L) over 40 s. Inset shows changes in the normalized emission spectrum upon irradiation at 254 nm in hexane (1 × 10^−5^ mol/L) over 40 s. (**b**) Absorbance decay of ***ap*-BN-DAE** at 475 nm at 25 °C in hexane (5 × 10^−5^ mol/L) after irradiation at 254 nm for 40 s. Inset shows absorbance changes of ***ap*-BN-DAE** at 475 nm upon irradiation with alternate lights (254 nm and 400–800 nm) over twenty cycles in hexane (5 × 10^−5^ mol/L) at 25 °C. (**c**) Changes in the absorption spectrum of ***p*-BN-DAE** upon irradiation at 254 nm in hexane (5 × 10^−5^ mol/L) over 40 s. Inset shows changes in the normalized emission spectrum upon irradiation at 254 nm in hexane (1 × 10^−5^ mol/L) over 40 s. (**d**) Changes in the absorption spectrum of **CC-DAE** upon irradiation at 365 nm in hexane (5 × 10^−5^ mol/L) over 40 s. Inset shows changes in the normalized emission spectrum upon irradiation at 365 nm in hexane (1 × 10^−5^ mol/L) over 40 s.

**Figure 2:**
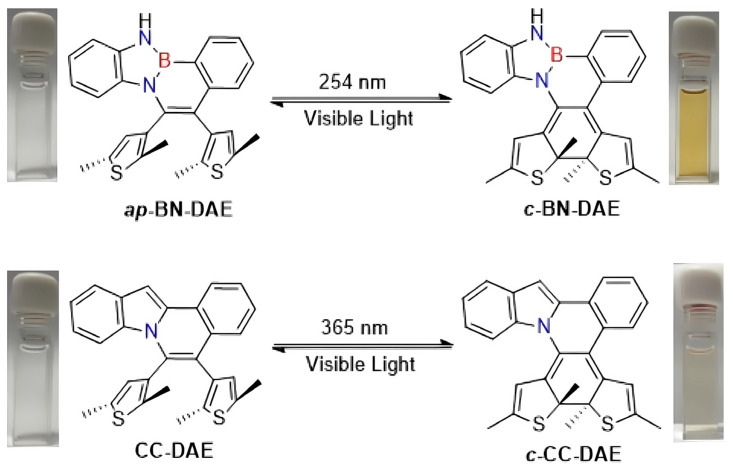
Color changes of ***ap*-BN-DAE** and **CC-DAE** in hexane (5 × 10^−5^ mol/L).

The photochromism of ***p*-BN-DAE**, **a*p*-BN-DAE** and **CC-DAE** was then studied. Figure 1a shows the photoconversion of **a*p*-BN-DAE**. The absorption spectrum of ***ap***-**BN-DAE** in hexane (5 × 10^−5^ M) shows a lowest-energy peak at 340 nm. Following irradiation at 254 nm, the appearance of a low-energy band at ∼475 nm and five isosbestic points (∼273, 289, 292, 299, and 310 nm) was observed, accompanied by the transformation of the colorless solution to yellow (Figure 2). The significant difference mainly results from rearranged *π*-electrons, caused by photocyclization [25]. The photoreaction proceeds to a photostationary state within 40 s. Subsequent irradiation with white light for 4 min completely regenerates the open form from the closed form. ***ap*-BN-DAE** exhibits a fluorescent emission maximum at ∼392 nm upon excitation at its isosbestic point (292 nm). During photoconversion, fluorescence is quenched, and the photoluminescence quantum yield (PLQY) decreases from 1.17% to 0.04% (Figures S10 and S11). We further evaluated the fatigue resistance and thermodynamic stability of ***ap*-BN-DAE** (Figure 1b). ***ap*-BN-DAE** displayed a moderate fatigue resistance, undergoing fully reversible photochemical ring-opening and ring-closing processes in hexane for more than twenty cycles with 24% degradation. Meanwhile, the closed form showed a high thermodynamic stability at 298 K, exhibiting no significant degradation after 15 days when stored sealed, in the dark. In addition, the photochromic behavior of ***ap*-BN-DAE** was investigated in various solvents, including benzene, dichloromethane, dimethyl sulfoxide and methanol (Figures S6–S9). The results revealed that the photochromism of ***ap*-BN-DAE** exhibits a strong solvent dependence: in polar solvents, the process is photoirreversible, whereas in less polar benzene, reaching the photostationary state required two minutes—significantly slower than in hexane. The photochromism of ***p*-BN-DAE** was also investigated despite the theoretical prohibition of 6π-electron photocyclization for parallel-configured diarylethenes under the Woodward–Hoffmann rules. In contrast to the hindered non-photoresponsive *p*-conformer of a reported system [24], ***p*-BN-DAE** exhibits clear photochromic behavior (Figure 1c). Nevertheless, its initial photocyclization rate is substantially lower than that of **a*p*-BN-DAE**. A plausible explanation is that irradiation promotes interconversion between conformers by enabling the system to cross the rotation energy barrier between the *p*- and *ap*-conformers, confirmed by the following NMR experiments and theoretical calculations. Furthermore, ***p*-BN-DAE** shows a higher PLQY (4.42%), which is consistent with its observed slower rate of photocyclization relative to the *ap*-conformer. Figure 1d shows the photoconversion of **CC-DAE**. Upon irradiation with 365 nm light, **CC-DAE** exhibited no marked photoresponsive change, characterized by the appearance of a low-energy band at ~477 nm and an isosbestic point at ~307 nm, without a perceptible change in solution color (Figure 2). Notably, while photochromism of **CC-DAE** also occurs under 254 nm light irradiation, the photocyclization rate is significantly lower than that observed with 365 nm irradiation. The photocyclization quantum yield of **a*p*-BN-DAE** was determined using BTF_6_ as a reference [24]. An excellent photocyclization quantum yield of 89.2% was obtained, which exceeds those of most reported diarylethene systems [39,40,41].

**Figure 3:**
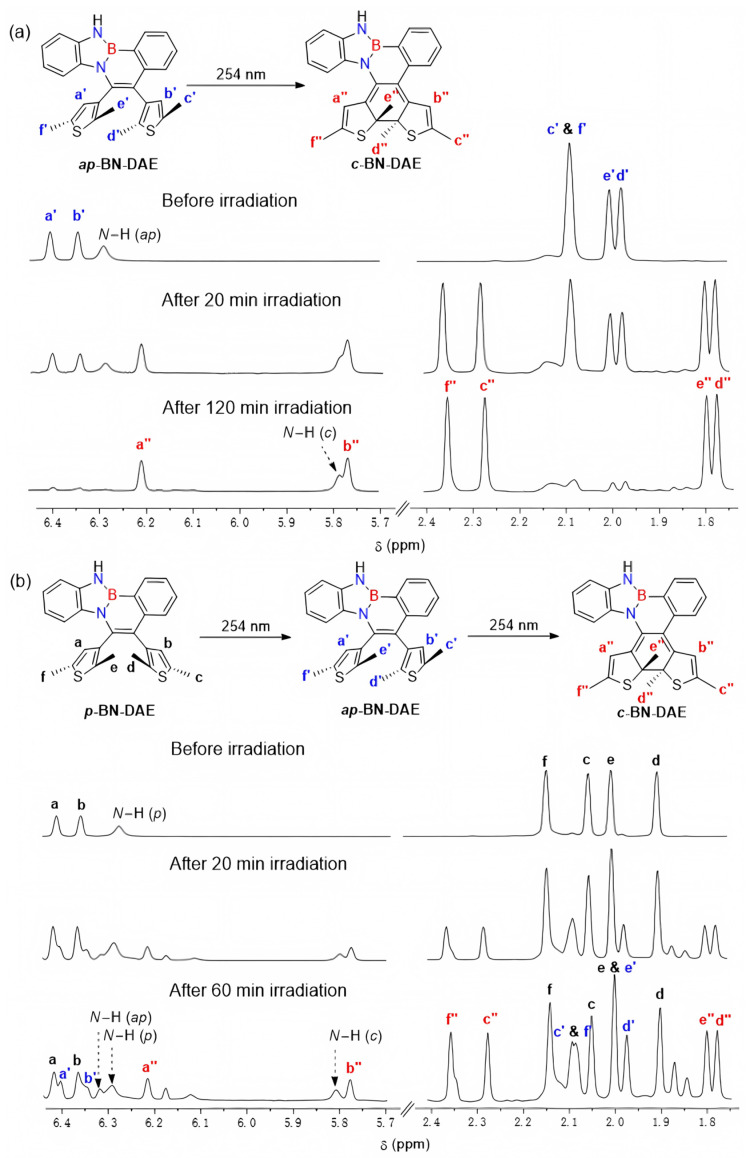
(**a**) Partial changes in the ^1^H NMR signals of ***ap*-BN-DAE** upon 254 nm irradiation in C_6_D_6_ (2.3 × 10^−3^ mol/L, detected with a quartz NMR tube); (**b**) Partial changes in the ^1^H NMR signals of ***p*-BN-DAE** upon 254 nm irradiation in C_6_D_6_ (2.3 × 10^−3^ mol/L, detected with a quartz NMR tube).

The photochromism of ***ap*-BN-DAE** and ***p*-BN-DAE** in C_6_D_6_ was further monitored by ^1^H NMR spectroscopy (Figure 3). Herein, we only present the chemical shifts of dimethylthienyl fragments for clarity. As depicted in Figure 3a, the signals at δ = 6.41 and 6.35 ppm correspond to the protons adjacent to the thiophene rings, the methyl protons appear as only three signals at δ = 2.09, 2.00 and 1.98 ppm, and the signal at δ = 2.09 ppm corresponds to two methyl groups. Upon irradiation at 254 nm for 20 min, approximately 57% of ***ap*-BN-DAE** underwent conversion to the closed form, ***c*-BN-DAE**. This transformation was evidenced by an upfield shift in the thienyl proton signals to 6.21 and 5.77 ppm, as well as new methyl proton signals at 2.35, 2.27, 1.79, and 1.77 ppm. Extending the irradiation to 120 min increased the conversion yield to 94%, which was related to the first open-to-closed switching step. During the photocyclization process, no signal for interconverted ***p*-BN-DAE** was observed. Comparatively, in Figure 3b, the ^1^H NMR spectrum of ***p*-BN-DAE** displays two thienyl proton signals at δ 6.42 and 6.37 ppm, and four methyl proton signals at δ 2.14, 2.06, 2.00, and 1.90 ppm. Irradiation for 20 min generated new signals corresponding to **a*p*-BN-DAE** and ***c*-BN-DAE**. Prolonged irradiation for 60 min resulted in a 26% conversion to the closed form, albeit with concurrent formation of the side product. The overall conversion rate of **BN-DAE** to the closed form was determined to be 65%. These experimental results revealed that the photochromism of ***p*-BN-DAE** proceeds via an interconversion from the *p*-conformer to the *ap*-conformer.

**Figure 4:**
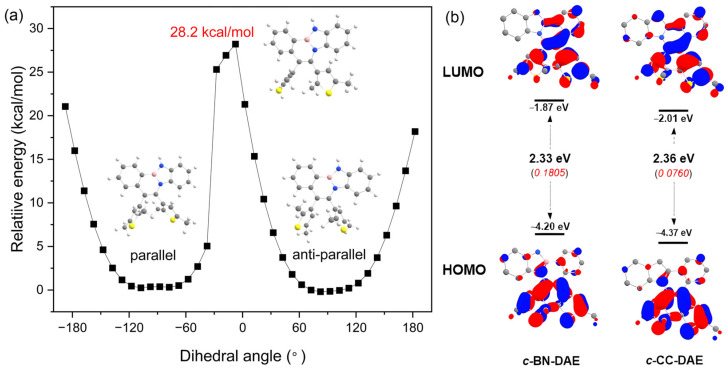
(**a**) Potential energy surface of ***p*-BN-DAE** with respect to rotation of dihedral angle [−180° to 180°], calculated at the B3LYP/6-31G(d,p) level of theory. Note: at each step, the dihedral angle was constrained while all other geometric parameters were fully optimized. (**b**) Spatial plots (isovalue = 0.03) of HOMO and LUMO, energy (in eV), HOMO-LUMO gap and oscillator strength (red) for the closed form of **BN-DAE** and **CC-DAE**.

To probe the interconversion between the parallel and *anti*-parallel conformations of **BN-DAE**, a relaxed potential energy surface (PES) scan of the parallel conformer was performed at the B3LYP/6-31G(d,p) level of theory. The scan was conducted along the key dihedral angle connecting the two thiophene units over the range of −180° to 180° in 10° increments (Figure 4a). The relaxed PES scan reveals two well-defined minima corresponding to the parallel and *anti*-parallel conformations, separated by a rotational barrier of 28.2 kcal/mol. This large barrier renders thermal interconversion kinetically inaccessible under ambient temperature, consistent with the absence of spontaneous conformational exchange in the proton NMR spectra. To gain deeper insight into the differences in electronic structure between **BN-DAE** and **CC-DAE**, density functional theory (DFT) and time-dependent DFT (TD-DFT) calculations were performed. As shown in Figure 4b, the highest occupied molecular orbital (HOMO) and the lowest unoccupied molecular orbital (LUMO) of the closed form of **BN-DAE** and **CC-DAE** exhibit high similarity in both energy and distribution. Furthermore, the closed form of **BN-DAE** possesses a larger oscillator strength for the first excited state—primarily contributed by the HOMO→LUMO transition—compared to that of **CC-DAE**. This result is consistent with its more intense absorption at 475 nm and explains the more distinct color change of ***ap*-BN-DAE** upon irradiation.

## 3. Conclusions

In summary, we have developed a high-performance photochromic system based on a boron–nitrogen heterocycle-bridged diarylethene (**BN-DAE**). Comparative analysis with its carbonaceous counterpart (**CC-DAE**) underscores the efficacy of BN/CC isosterism as a design strategy. Following separation of the parallel and *anti*-parallel conformers, detailed photochromic evaluation identified ***ap*-BN-DAE** as the superior isomer, combining a high photoconversion quantum yield, moderate fatigue resistance in hexane, and high thermodynamic stability of its closed form at 298 K. A rotation energy barrier of 28.2 kcal/mol between the *ap*- and *p*-conformers was determined by a potential energy surface (PES) scan, making interconversion inaccessible under ambient conditions. Proton NMR spectroscopy showed that upon irradiation, ***p*-BN-DAE** can overcome this barrier to convert to the *ap*-conformer. Conversely, **CC-DAE** exhibited negligible photochromism in solution, with only a faint color change upon UV irradiation. DFT and TD-DFT calculations reveal that the closed form of **BN-DAE** exhibits a higher oscillator strength than its carbonaceous counterpart, consistent with its more intense UV absorption. These results demonstrate that BN/CC isosterism is a powerful strategy for optimizing the photochromic properties of diarylethenes and pave the way for the rational design of advanced photochromic materials through strategic heteroatom substitution.

## Data Availability

Data are available upon request.

## Supplementary Materials

The following supporting information can be downloaded at: https://www.mdpi.com/article/10.3390/molecules31071115/s1, including reagents, characterization, synthesis details, photochromic properties, crystallographic, NMR, HRMS spectra and excitation calculation, and cartesian coordinates.
