# Supplementary material for: High-Performance Photochromic Diarylethene with a Boron–Nitrogen Heterocyclic Ethene Bridge
Source: Molecules. 2026 Mar 28;31(7):1115. doi: 10.3390/molecules31071115 (PMC13074988; doi:10.3390/molecules31071115)
Supplement: Supplementary file 1 [file molecules-31-01115-s001.zip › molecules-4219531-supplementary.pdf]

Supplementary Information

High-Performance Photochromic Diarylethene with a Boron-Nitrogen-Heterocyclic Ethene Bridge

Chen Zhang [1], Yuping Dai [2], Yilin Chen [1], Shaoqiang Dong [3] and Jiaxing Wang [1]*

[1.] School of Materials Science and Engineering, Dongguan University of Technology, Dongguan 523808, China.

[2.] Institut des Sciences Analytiques et de Physico-Chimie pour l'Environnement et les Matériaux IPREM UMR 5254, Université de Pau et des Pays de l'Adour, 64053 Pau, France.

[3.] State Key Laboratory of Advanced Materials for Intelligent Sensing and Key Laboratory of Organic Integrated Circuits, Ministry of Education & Tianjin Key Laboratory of Molecular Optoelectronic Sciences, Department of Chemistry, Institute of Molecular Aggregation Science, Tianjin University, No. 92 Weijin Road, Nankai District, Tianjin 300072, China.

General

Reagents. 2,5-Dimethylthiophene, potassium carbonate, bis(triphenylphosphine)palladium(II) chloride, triethylamine (extra dry, with molecular sieves), *o*-phenylenediamine, molecular sieve 3A, sodium carbonate, copper (II) acetate monohydrate, tetrahydrofuran (extra dry, with molecular sieves), *o*-xylene (extra dry, with molecular sieves), cyclopentyl methyl ether (extra dry, with molecular sieves), palladium (II) acetate, triphenylphosphine and lithium chloride were purchased from Energy Chemical (Shanghai, China). *N*-iodosuccinimide, cuprous iodide, 2-bromophenylboronic acid, trimethylsilylacetylene and (pentamethylcyclopentadienyl)rhodium (III) dichloride dimer were purchased from Tansoole (Shanghai, China). All chemicals were used as received. All air-sensitive manipulations were carried out under an inert atmosphere using standard Schlenk techniques.

Characterization. NMR spectra were recorded on Bruker Ascend-400 spectrometer at 400 MHz (^{1}H NMR), 101 MHz (^{13}C NMR), 128 MHz (^{11}B NMR). The residual solvent signals were used as references for ^{1}H and ^{13}C NMR spectra and the chemical shifts converted to the TMS scale ($CDCl_3$: δH = 7.26 ppm, δC = 77.16 ppm; C_6D_6: δH = 7.16 ppm). The chemical shifts of ^{11}B NMR spectra were against BF_3•OEt_2 (δB = 0 ppm). Multiplicities of ^{1}H NMR are reported as follows: singlet (s), doublet (d), triplet (t), doublet of doublets (dd), multiplet (m), and broad resonance (br). High resolution mass spectra (HRMS) were recorded on an Agilent 6540TOF LC/MS with Electron Spray Ionization (ESI) resource. Single crystal structure data were obtained with the Bruker Kappa APEX DUO diffractometer. Analytic HPLC was performed by using a SHIMADZU LCMS-2020 instrument equipped with C18 column (4.6 × 250 mm, Shim-pack GIST). Preparative HPLC were performed by using a SHIMADZU LCMS-2020 instrument equipped with C18 column (20 × 50 mm, Shim-pack GIST (G)). UV-Vis spectra were recorded with HITACHI UH4150 spectrometers. Fluorescent spectra were recorded on an Edinburgh Instruments FLS 1000 spectrometer. Solution quantum yields were collected with a calibrated integrating sphere system. The photocyclization

reaction was induced *in situ* by continuous irradiation using a hand Hg lamp (Ji Hui Analysis Instrument (Shanghai) CO., LTD, ZF-7A, 254 nm, 16 W), the photocycloreversion reaction was induced *in situ* by continuous irradiation using a hand LED lamp (common commercially available product, 5 W). The photochromic reaction quantum yields were evaluated by the standard procedures using BTF6 as the references for photocyclization and photocycloreversion.

Synthetic Procedures

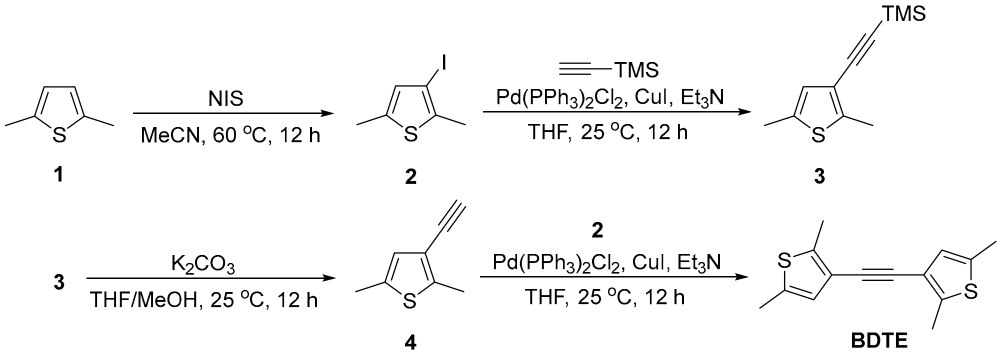

Scheme S1. Synthetic route of **BDTE**.

3-Iodo-2,5-dimethylthiophene (***2***). A solution of *N*-iodosuccinimide (22.5 g, 100 mmol, 1.00 equiv.) in 300 mL acetonitrile was added into a solution of 2,5-dimethylthiophene (11.2 g, 100 mmol, 1.00 equiv.) in 100 mL acetonitrile. The reaction mixture was heated up to 60 °C and stirred at dark place for 12 hours. After removal of solvents under reduced pressure, the residue was redissolved into 300 mL water followed by addition of 20 mL saturated sodium thiosulfate aqueous solution. The mixture was extracted with 80 mL hexane three times. After concentration of the combined organic phase, the crude product was purified by column chromatography on silica gel (using hexane as the eluent) to afford the product as a colorless oil (19.3 g, 81%). ^{1}H NMR (400 MHz, $CDCl_3$): δ 6.61 (s, 1H), 2.41 (s, 3H), 2.35 (s, 3H). The proton NMR is identical to the reported data. [S1]

((2,5-Dimethylthiophen-3-yl)ethynyl)trimethylsilane (***3***). $Pd(PPh_3)_2Cl_2$ (1.75 g, 2.50 mmol, 0.05 equiv.), CuI (476 mg, 2.50 mmol, 0.05 equiv.) were suspended in 150 mL anhydrous tetrahydrofuran under nitrogen atmosphere. To the mixture were added 3-iodo-2,5-dimethylthiophene (**2**, 11.9 g, 50.0 mmol, 1.00 equiv.), triethylamine (27.8 mL, 200 mmol, 4.00 equiv.) and trimethylsilylacethylene (8.36 mL, 60.0 mmol, 1.20 equiv.) *via* syringe. After stirring at 25 °C for 12 hours, the reaction mixture was concentrated *via* rotary evaporator. The residue was suspended in 200 mL hexane *via* ultrasound and passed through a filter paper. After concentration of the filtrate, the crude product was purified *via* silica gel chromatography, using hexane as the eluent, to afford the product as a colorless oil (9.69 g, 93%). ^{1}H NMR (400 MHz, $CDCl_3$) δ 6.60 (s, 1H), 2.44 (s, 3H), 2.36 (s, 3H), 0.23 (s, 9H). The proton NMR is identical to the reported data. [S1]

3-Ethynyl-2,5-dimethylthiophene (***4***). To a solution of ((2,5-dimethylthiophen-3-yl)ethynyl)trimethylsilane (**3**, 8.34 g, 40.0 mmol, 1.00 equiv.) in mixed solvent THF/MeOH (200 mL, 3:1 v/v) was added anhydrous potassium carbonate (22.1 g, 160 mmol, 4.00 equiv.). The reaction mixture was allowed to stir at 25 °C for 12 hours. After removal of solvent under reduced

pressure, the residue was suspended in 200 mL hexane *via* ultrasound and passed through a filter paper. After concentration of the filtrate, the crude product was purified *via* silica gel chromatography, using hexane as the eluent, to afford the product as a colorless oil (4.58 g, 84%). ^{1}H NMR (400 MHz, $CDCl_3$) δ 6.63 (s, 1H), 3.13 (s, 1H) 2.47 (s, 3H), 2.38 (s, 3H). The proton NMR is identical to the reported data. [S1]

1,2-Bis(2,5-dimethylthiophen-3-yl)ethyne (***BDTE***). Under nitrogen atmosphere, a 250 mL oven-dried, round-bottomed flask was charged with 3-ethynyl-2,5-dimethylthiophene (**4**, 4.09 g, 30.0 mmol, 1.00 equiv.), 3-iodo-2,5-dimethylthiophene (**2**, 8.57 g, 36.0 mmol, 1.20 equiv.), $Pd(PPh_3)_2Cl_2$ (1.05 g, 1.50 mmol, 0.05 equiv.), CuI (286 mg, 1.50 mmol, 0.05 equiv.) triethylamine (10.7 mL, 120 mmol, 4.00 equiv.) and 80 mL anhydrous tetrahydrofuran. After stirring at 25 °C for 12 hours, the reaction mixture was concentrated under reduced pressure. The residue was suspended in 150 mL hexane *via* ultrasound and passed through a filter paper. After concentration of the filtrate, the crude product purified *via* silica gel chromatography, using hexane as the eluent, to afford the product as a pale-yellow solid (6.58 g, 89%). ^{1}H NMR (400 MHz, $CDCl_3$) δ 6.65 (s, 2H), 2.49 (s, 6H), 2.39 (s, 6H). The proton NMR is identical to the reported data. [S1]

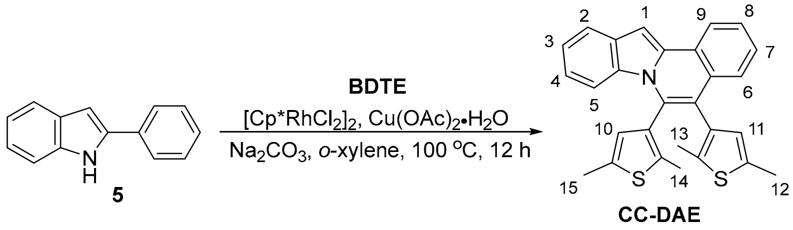

Scheme S2. Synthesis of **CC-DAE**.

5,6-Bis(2,5-dimethylthiophen-3-yl)indolo[2,1-a]isoquinoline (***CC-DAE***). Under nitrogen atmosphere, a 10 mL Schlenk tube was charged with 2-phenyl-1*H*-indole (**5**, 193 mg, 1.00 mmol, 1.00 equiv.), 1,2-bis(2,5-dimethylthiophen-3-yl)ethyne (**BDTE**, 246 mg, 1.00 mmol, 1.00 equiv.), (pentamethylcyclopentadienyl)rhodium(III) dichloride dimer (**$[Cp^*RhCl_2]_2$**, 12.5 mg, 0.02 mmol, 0.02 equiv.), copper (II) acetate monohydrate (20.0 mg, 0.1 mmol, 0.1 equiv.), anhydrous sodium carbonate (212 mg. 2.00 mmol, 2.00 equiv.) and 4 mL anhydrous *o*-xylene. After stirring at 100 °C for 12 hours, the reaction mixture was diluted with 20 mL hexane and passed through a filter paper. After concentration of the filtrate, the residue was purified *via* silica gel chromatography (using hexane-dichloromethane (10:1 v/v) as the eluent) to afford the product as a pale-yellow solid (298 mg, 68%). ^{1}H NMR (400 MHz, $CDCl_3$) δ 8.27 (d, J = 8.0 Hz, 2H, *H9*), 7.80 (d, J = 8.0 Hz, 2H, *H2*), 7.50 (t, J = 8.0 Hz, 2H, *H8*), 7.39 (dd, J_1 = 12.0 Hz, J_2 = 8.0 Hz, 2H, *H7*), 7.30-7.22 (m, 5H, *H1, H3, H6(parallel conformer)*), 7.19 (d, J = 8.0 Hz, 1H, *H6 (antiparallel conformer)*), 7.00 (t, J = 8.0 Hz, 2H, *H4*), 6.51 (s, 1H, *H10 (parallel conformer)*), 6.48 (s, 1H, *H10 (antiparallel conformer)*), 6.45 (d, J = 8.0 Hz, 1H, *H5 (parallel conformer)*), 6.40 (s, 1H, *H11 (antiparallel conformer)*), 6.37 (d, J = 8.0 Hz, 1H, *H5 (antiparallel conformer)*), 6.31 (s, 1H, *H11 (parallel conformer)*), 2.44 (s, 3H, *H15 (parallel conformer)*), 2.42 (s, 3H, *H15 (antiparallel conformer)*), 2.39 (s, 3H, *H12 (parallel conformer)*), 2.37 (s, 3H, *H12 (antiparallel conformer)*), 2.15-2.07 (m, 9H, H14, H13 *(antiparallel conformer)*), 2.00 (s, 3H, *H13 (parallel conformer)*). ^{13}C NMR (101 MHz, $CDCl_3$) δ 136.93, 136.79, 136.53, 136.23, 135.16, 135.01, 134.41, 132.99, 132.80, 132.74, 132.73, 132.20,

131.83, 129.65, 129.60, 128.92, 127.83, 127.71, 127.65, 127.51, 127.21, 126.30, 125.88, 125.83, 125.54, 125.51, 123.52, 123.44, 121.88, 120.94, 120.87, 120.23, 117.12, 114.03, 113.93, 15.48, 15.43, 15.40, 15.37, 14.32, 14.15, 14.00, 13.81. HRMS (ESI positive ion mode for $[M + H]^+$): Calcd for $C_{28}H_{23}NS_2$, 438.1350; found, 438.1353.

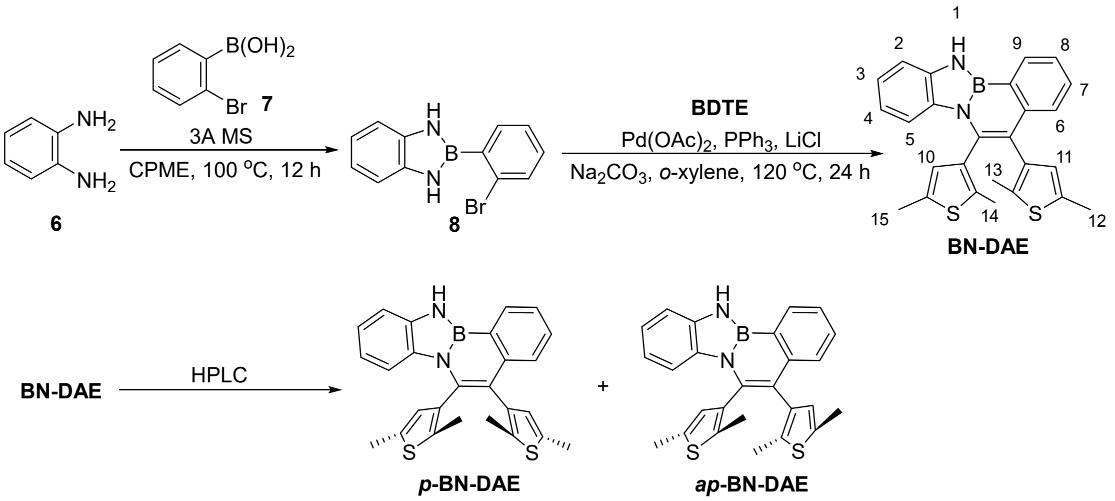

Scheme S3. Synthetic route of **BN-DAE**.

2-(2-Bromophenyl)-2,3-dihydro-1H-benzo[d][1,3,2]diazaborole (***8***). A 50 mL Schlenk tube was charged with (2-bromophenyl)boronic acid (**7**, 1.00 g, 5.00 mmol, 1.00 equiv.), benzene-1,2-diamine (**6**, 538 mg, 5.00 mmol, 1.00 equiv.), 500 mg 3A molecular sieve and 10 mL anhydrous cyclopentyl methyl ether (CPME). After stirring at 100 °C for 12 hours, the reaction mixture was diluted with 20 mL hexane and passed through a filter paper. After concentration of the filtrate, the residue was purified *via* silica gel chromatography (using hexane-ethyl acetate (10:1 v/v) as the eluent) to afford the product as a white powder (1.19 g, 87%). ^{1}H NMR (400 MHz, $CDCl_3$) δ 7.70-7.60 (m, 2H), 7.37 (t, J = 8.0 Hz, 1H), 7.31-7.23 (m, 1H), 7.20 (m, 2H), 7.06 (br, 2H), 7.04-6.95 (m, 2H). The proton NMR is identical to the reported data. [S2]

5,6-Bis(2,5-dimethylthiophen-3-yl)-12H -benzo[c]benzo[4,5][1,3,2]diazaborolo[1,2a][1,2]aza-borinine (***BN-DAE***). Under nitrogen atmosphere, a 50 mL Schlenk tube was charged with compound **8** (273 mg, 1.00 mmol, 1.00 equiv.), **BDTE** (493 mg, 1.50 mmol, 1.50 equiv.), $Pd(OAc)_2$ (22 mg, 0.1 mmol, 0.1 equiv.), triphenylphosphine (53 mg, 0.2 mmol, 0.2 equiv.), lithium chloride (42 mg, 1.00 mmol, 1.00 equiv.), sodium carbonate (212 mg, 2.00 mmol, 2.00 equiv.) and 10 mL anhydrous *o*-xylene. After stirring at 120 °C for 24 hours, the reaction mixture was diluted with 40 mL hexane and passed through a filter paper. After concentration of the filtrate, the residue was purified *via* silica gel chromatography (using hexane-dichloromethane (10:1 v/v) as the eluent) to afford the product as a white solid (242 mg, 55%). Parallel (*p*-) and antiparallel (a*p*-) conformers with a ratio of 43:57 were isolated *via* High-Performance Liquid Chromatography (HPLC).

***p*-BN-DAE**. ^{1}H NMR (400 MHz, $CDCl_3$) δ 8.22 (d, J = 8.0 Hz, 1H, *H9*), 7.55 (t, J = 8.0 Hz, 1H, *H7*), 7.45 (t, J = 8.0 Hz, 1H, *H8*), 7.37 (d, J = 8.0 Hz, 1H, *H2*), 7.29 (d, J = 8.0 Hz, 1H, *H6*), 7.20 (br, 1 H, *H1*), 7.10 (t, J = 8.0 Hz, 1H, *H3*), 6.82 (t, J = 8.0 Hz, 1H, *H4*), 6.46 (s, 1H, *H10*), 6.33 (s, 1H, *H11*), 6.28 (d, J = 8.0 Hz, 1H, *H5*), 2.41 (s, 3H, *H15*) , 2.37 (s, 3H, *H12*) , 2.04 (s, 3H, *H14*) ,

2.02 (s, 3H, *H13*). ^{1}H NMR (400 MHz, C_6D_6) δ 8.14 (d, *J* = 8.0 Hz, 1H), 7.70 (d, *J* = 8.0 Hz, 1H), 7.56-7.39 (m, 2H), 7.12 (d, *J* = 8.0 Hz, 1H), 7.04 (d, *J* = 8.0 Hz, 1H), 6.96 (t, *J* = 8.0 Hz, 1H), 6.66 (d, *J* = 8.0 Hz, 1H), 6.44 (s, 1H), 6.37 (s, 1H), 6.29 (br, 1H), 2.14 (s, 3H), 2.05 (s, 3H), 2.00 (s, 3H), 1.90 (s, 3H). ^{13}C NMR (101 MHz, $CDCl_3$) δ 141.22, 137.55, 135.92, 135.21, 134.87, 134.28, 134.07, 133.92, 133.91, 133.08, 131.35, 130.13, 129.59, 127.67, 126.43, 124.67, 121.70, 119.14, 117.57, 113.82, 111.97, 15.39, 15.35, 14.23, 13.83. (B-aryl carbon was not observed due to quadrupolar relaxation). ^{11}B NMR (128 MHz, $BF_3{\cdot}OEt_2$): δ 27.15. HRMS (ESI positive ion mode for $[M + H]^+$): Calcd for $C_{26}H_{23}BN_2S_2$, 439.1474; found, 439.1475.

***ap*-BN-DAE**. ^{1}H NMR (400 MHz, $CDCl_3$) δ 8.22 (d, *J* = 8.0 Hz, 1H, *H9*), 7.55 (t, *J* = 8.0 Hz, 1H, *H7*), 7.45 (t, *J* = 8.0 Hz, 1H, *H8*), 7.32 (d, *J* = 8.0 Hz, 1H, *H2*), 7.30 (d, *J* = 8.0 Hz, 1H, *H6*), 7.19 (br, 1 H, *H1*), 7.10 (t, *J* = 8.0 Hz, 1H, *H3*), 6.83 (t, *J* = 8.0 Hz, 1H, *H4*), 6.45 (s, 1H, *H10*), 6.37 (s, 1H, *H11*), 6.22 (d, *J* = 8.0 Hz, 1H, *H5*), 2.40 (s, 3H, *H15*) , 2.37 (s, 3H, *H12*) , 2.09 (s, 3H, *H14*) , 2.07 (s, 3H, *H3*). ^{1}H NMR (400 MHz, C_6D_6) δ 8.15 (d, *J* = 8.0 Hz, 1H), 7.67 (d, *J* = 8.0 Hz, 1H), 7.46 (t, *J* = 8.0 Hz, 2H), 7.08-6.90 (m, 2H), 6.61 (d, *J* = 8.0 Hz, 1H), 6.40 (s, 1H), 6.34 (s, 1H), 6.29 (br, 1H), 2.19-2.03 (m, 6H), 2.00 (s, 3H), 1.98 (s, 3H). ^{13}C NMR (101 MHz, $CDCl_3$) δ 141.54, 137.51, 136.14, 135.26, 134.92, 134.50, 134.32, 133.87, 133.84, 133.08, 131.31, 130.10, 128.39, 126.41, 126.34, 124.69, 121.71, 119.23, 117.40, 113.76, 111.98, 15.40 (two carbons), 14.12, 14.00. (B-aryl carbon was not observed due to quadrupolar relaxation). ^{11}B NMR (128 MHz, $BF_3{\cdot}OEt_2$): δ 27.06. HRMS (ESI positive ion mode for $[M + H]^+$): Calcd for $C_{26}H_{23}BN_2S_2$, 439.1474; found, 439.1475.

Table S1. Crystal data for **CC-DAE**.

Moiety formula	C28 H23 N S2 C28 H23 N S2	
Sum formula	C28 H23 N S2 C28 H23 N S2	
Formula weight	437.13	
Temperature	273 K	
Wavelength	1.54178 Å	
Space group	-P 2ybc	
Unit cell dimensions	a = 7.61600 Å	a= 90°.
	b = 31.23500 Å	b= 99.7300°.
	c = 20.52700 Å	g = 90°.
Volume	4812.839 Å3	
Z	8	
Density (calculated)	1.208 g/cm3	
Absorption coefficient	2.102 mm-1	
F(000)	1840.0	
h, k, lmax	9, 37, 24	
Nref	8600.	
Reflections collected	7790	
Independent reflections	7790 [R(int) = ?]	
Completeness to theta = 67.135°	98.8 %	
Absorption correction	Semi-empirical from equivalents	
Max. and min. transmission	0.845 and 0.777	

Refinement method	Full-matrix least-squares on F2
Data / restraints / parameters	7790 / 2 / 1155
Goodness-of-fit on F2	1.070
R indices (all data)	R1 = 0.1024, wR2 = 0.22920
Extinction coefficient	0.00030(9)

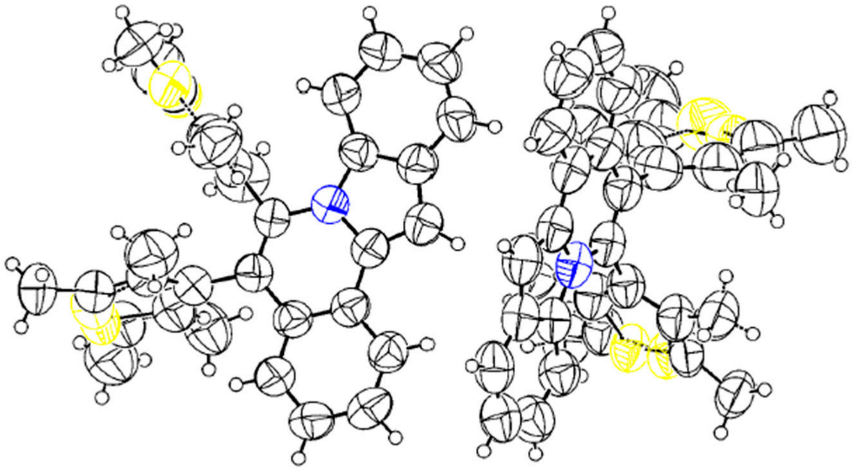

Figure S1. ORTEP representation of the crystal structure of CC-DAE with displacement ellipsoids shown at the 50 % probability level.

Photophysical and Photochromic Properties

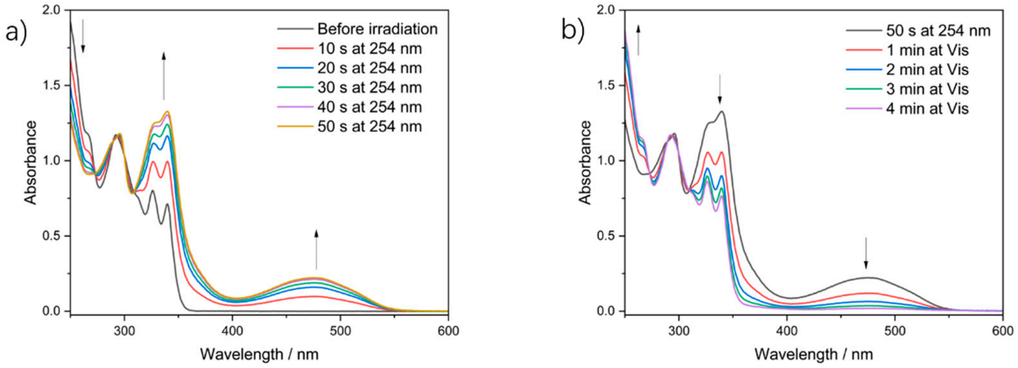

Figure S2. a) The photocyclization process of ***ap*-BN-DAE** in hexane (5 × 10^{-5} mol/L). b) The photocycloreversion process of ***ap*-BN-DAE** in hexane (5 × 10^{-5} mol/L).

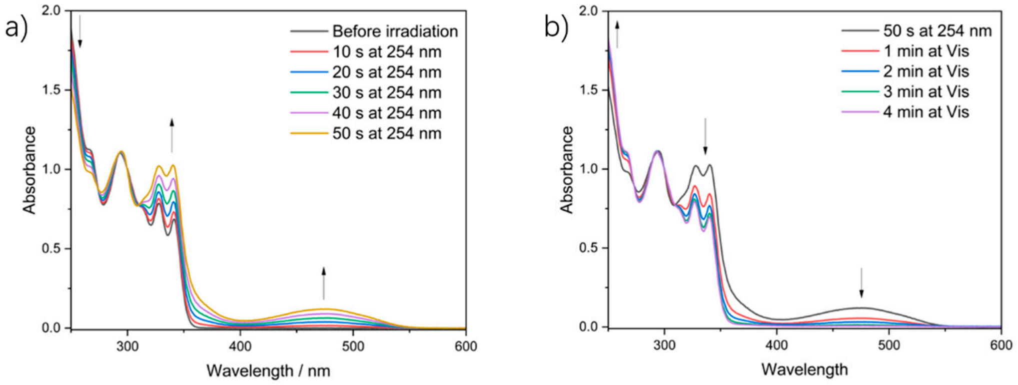

Figure S3. a) The photocyclization process of ***p*-BN-DAE** in hexane (5 × 10^{-5} mol/L). b) The photocycloreversion process of ***p*-BN-DAE** in hexane (5 × 10^{-5} mol/L).

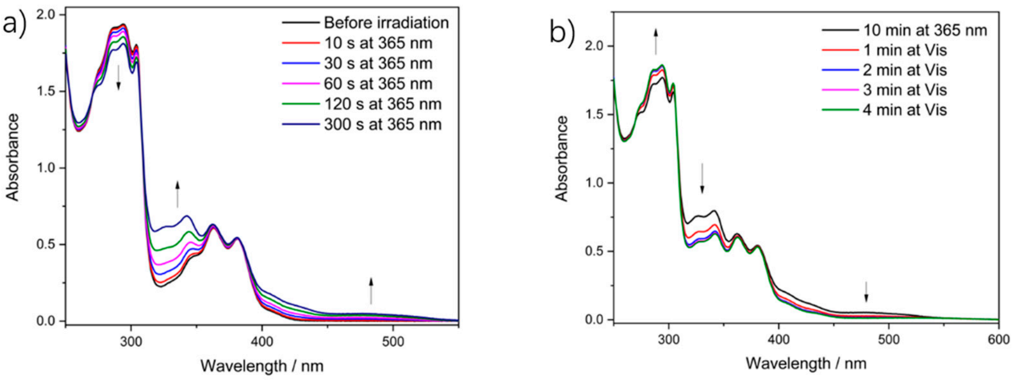

Figure S4. a) The photocyclization process of **CC-DAE** in hexane (5 × 10^{-5} mol/L). b) The photocycloreversion process of **CC-DAE** in hexane (5 × 10^{-5} mol/L).

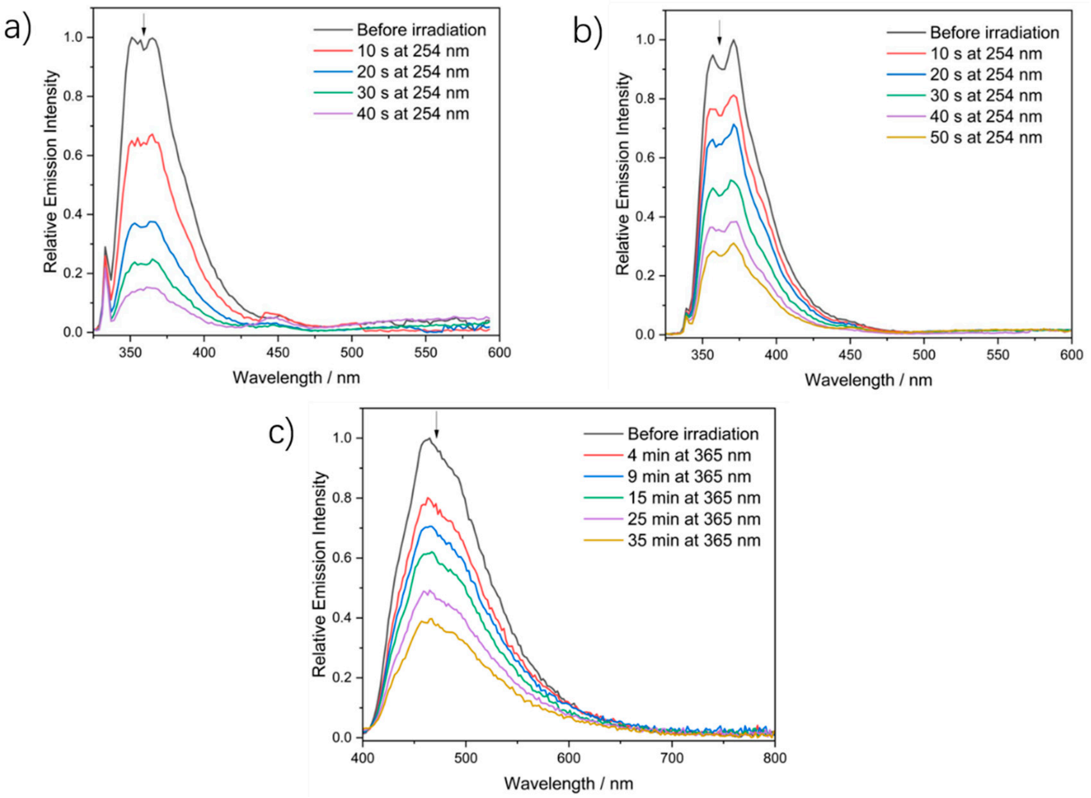

Figure S5. a) The normalized emission change in the photocyclization process of ***ap*-BN-DAE** in hexane (1 × 10^{-5} mol/L). b) The normalized emission change in the photocyclization process of ***p*-BN-DAE** in hexane (1 × 10^{-5} mol/L). c) The normalized emission change in the photocyclization process of **CC-DAE** in hexane (1 × 10^{-5} mol/L).

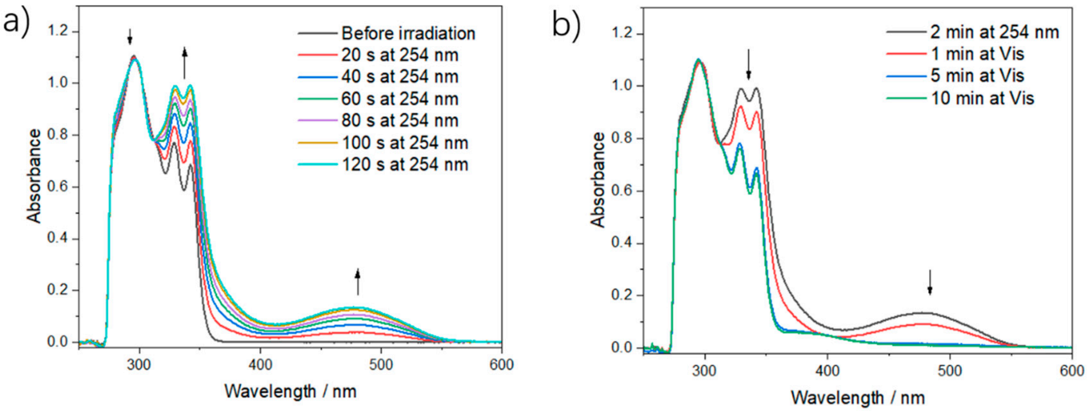

Figure S6. a) The photocyclization process of ***ap*-BN-DAE** in benzene (5 × 10^{-5} mol/L). b) The photocycloreversion process of ***ap*-BN-DAE** in benzene (5 × 10^{-5} mol/L).

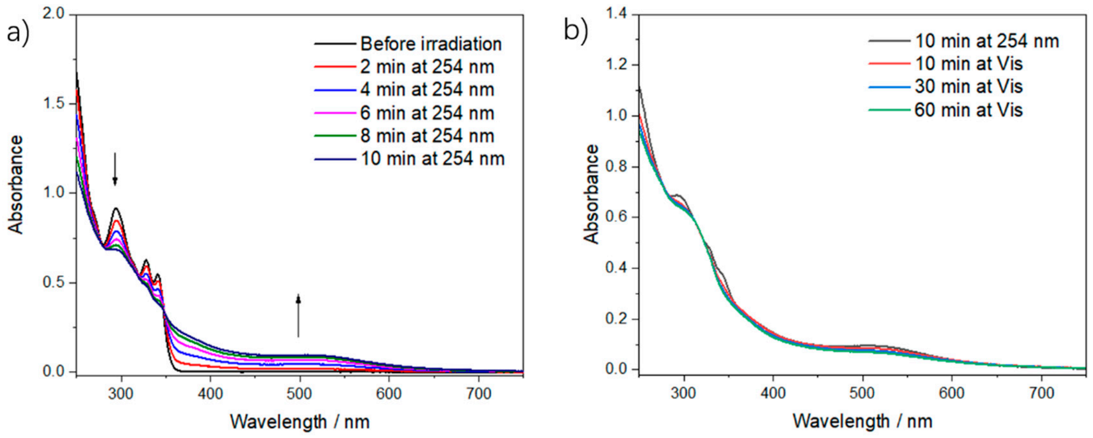

Figure S7. a) The photocyclization process of ***ap*-BN-DAE** in dichloromethane (5 × 10^{-5} mol/L). b) The photocycloreversion process of ***ap*-BN-DAE** in dichloromethane (5 × 10^{-5} mol/L). Note: ***ap*-BN-DAE** was not photocycloreversable in dichloromethane.

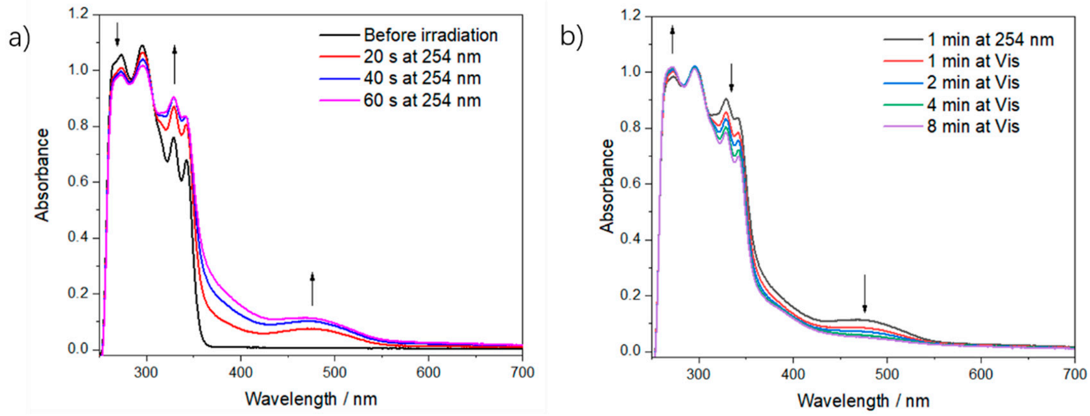

Figure S8. a) The photocyclization process of ***ap*-BN-DAE** in dimethyl sulfoxide (5 × 10^{-5} mol/L). b) The photocycloreversion process of ***ap*-BN-DAE** in dimethyl sulfoxide (5 × 10^{-5} mol/L).

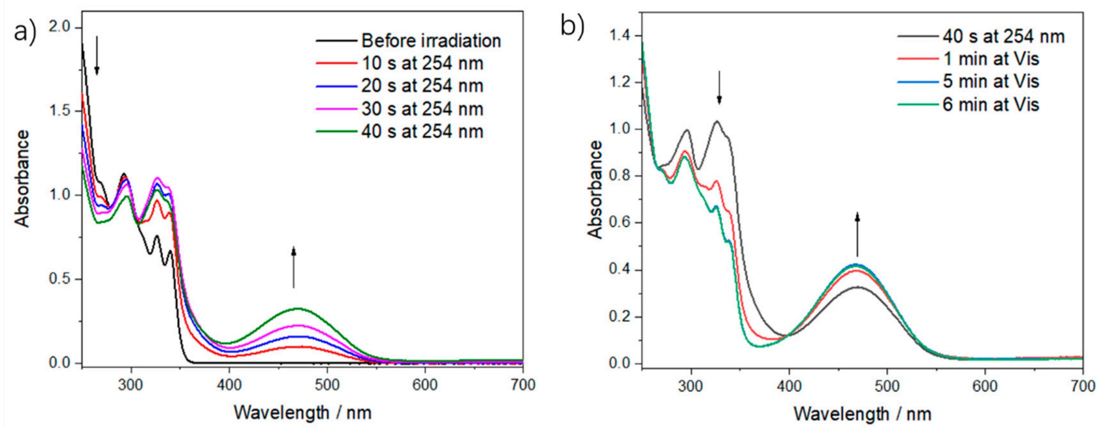

Figure S9. a) The photocyclization process of ***ap*-BN-DAE** in methanol (5 × 10^{-5} mol/L). b) The photocycloreversion process of ***ap*-BN-DAE** in methanol (5 × 10^{-5} mol/L). Note: ***ap*-BN-DAE** was not photocycloreversable in methanol.

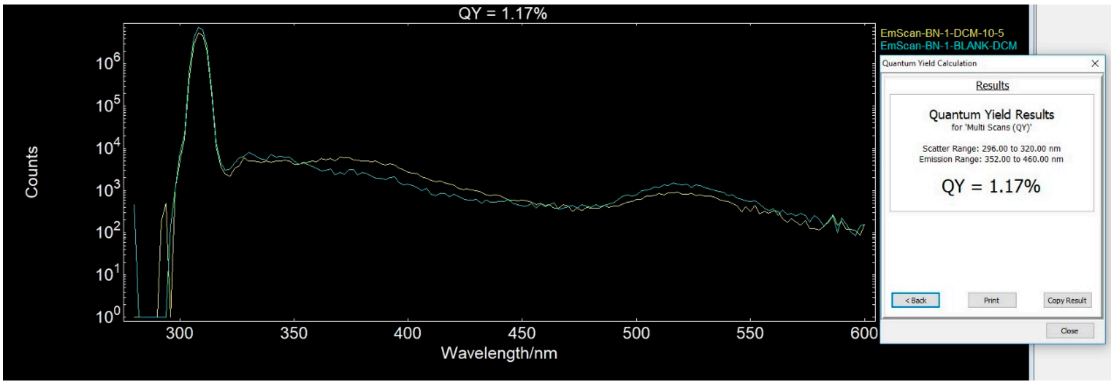

Figure S10. Photoluminescent quantum yield of ***ap*-BN-DAE**.

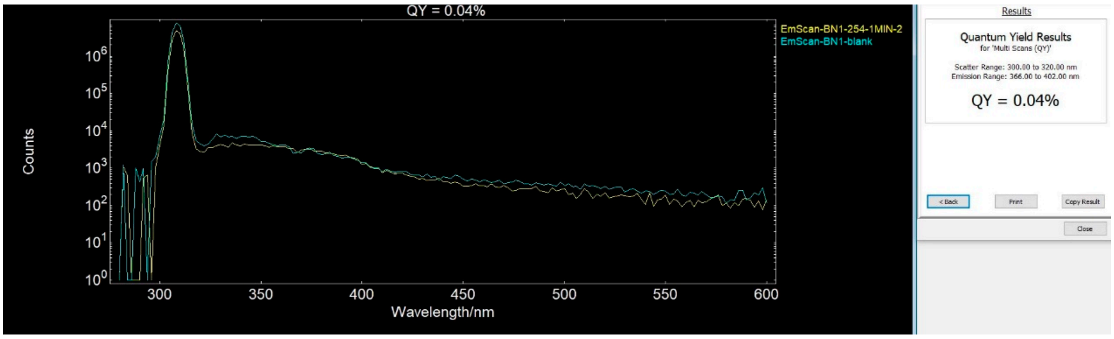

Figure S11. Photoluminescent quantum yield of ***ap*-BN-DAE** after irradiation.

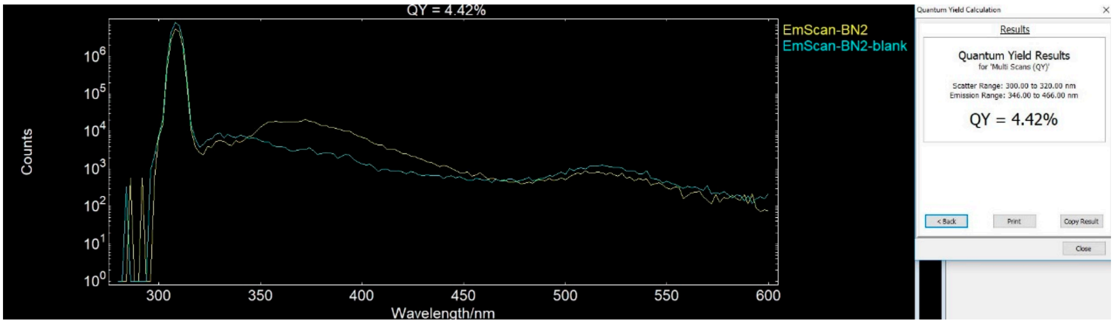

Figure S12. Photoluminescent quantum yield of ***p*-BN-DAE**.

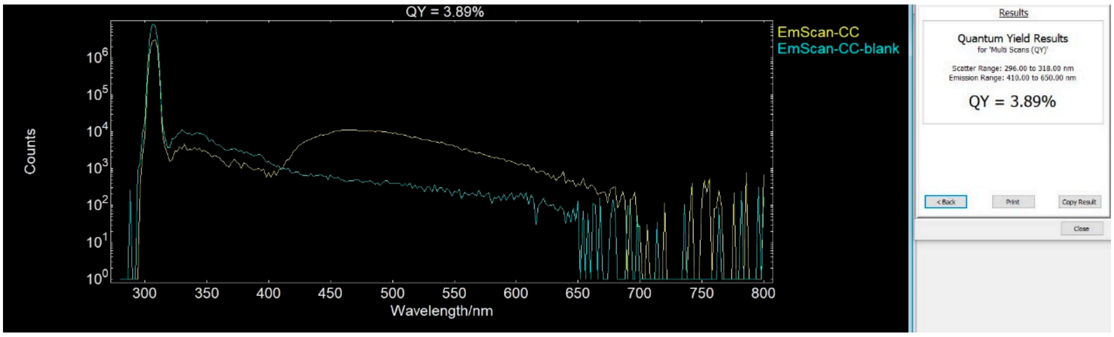

Figure S13. Photoluminescent quantum yield of **CC-DAE**.

Computational Details

All calculations were carried out using the Gaussian 16 program. The geometries were optimized at the B3LYP/6-31G(d,p) level. All positive vibrational frequencies were performed to ensure that the structures were minima. TD-DFT single-point calculations for vertical excitation were carried out at the B3LYP/6-31G(d,p) level. The empirical dispersion correction was added by using the "empiricaldispersion=gd3bj" keyword for all calculations.

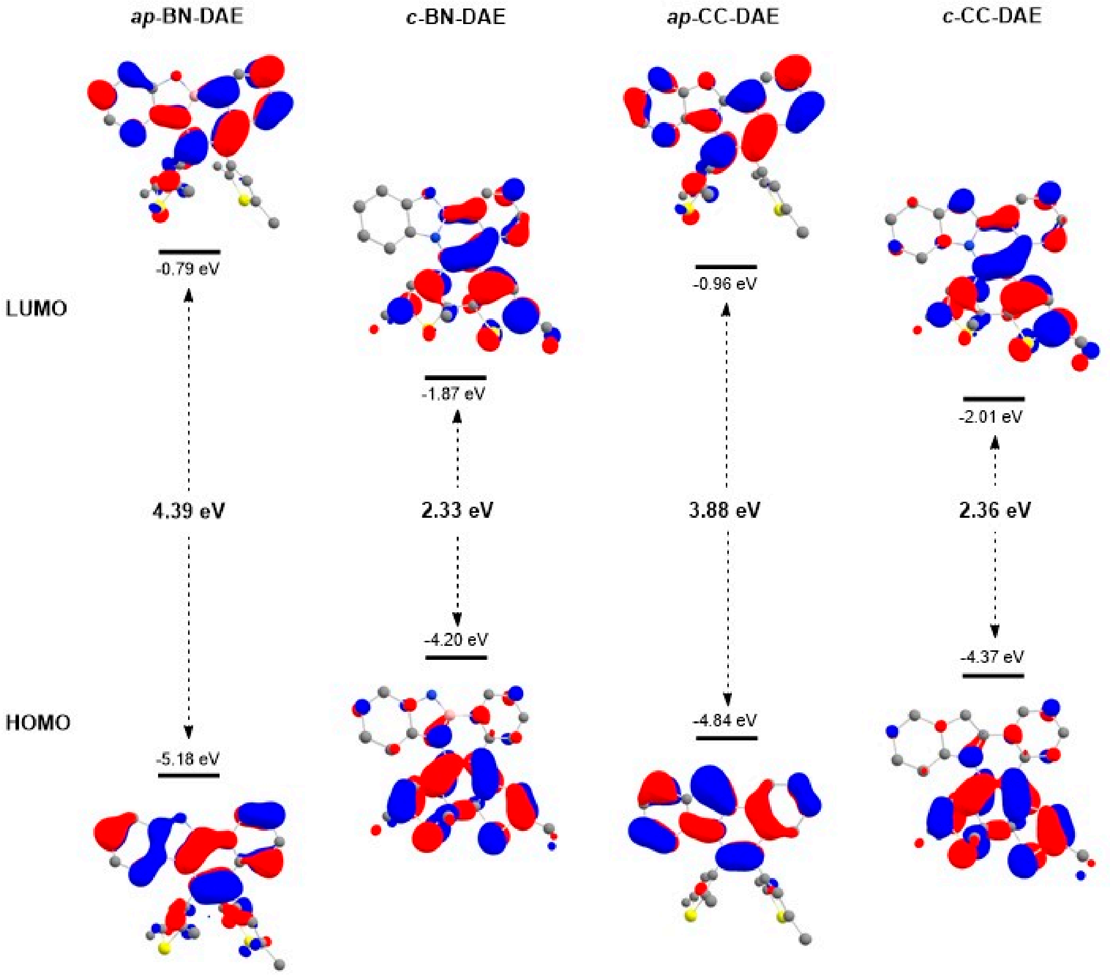

Figure S14. Molecular frontier orbital isosurfaces (isovalue = 0.03), energies (in eV) and HOMO-LUMO gap (in eV) of ***ap*-BN-DAE**, ***c*-BN-DAE**, ***ap*-CC-DAE** and ***c*-CC-DAE.**

Table S2. Vertical excitation wavelengths (nm) and energies (eV), oscillator strengths (f) and contributions of major molecular orbital transitions for molecules ***ap*-BN-DAE**, ***c*-BN-DAE**, ***ap*-CC-DAE**, ***c*-CC-DAE**, predicted by TD-DFT calculations. (Orbital contribution was calculated by (transition coefficient)^2 x 2 x 100%.

Excited state	λ, nm (eV)	Oscillator Strength, *f*	Major Orbital Transition
		***ap*-BN-DAE**	
S_1	319 (3.89)	0.0809	H→L (81%)
S_2	307 (4.04)	0.0041	H-1→L (48%), H→L+1 (45%)
S_3	297 (4.17)	0.0174	H-2→L (75%)
S_4	292 (4.25)	0.1502	H-1→L (44%), H→L+1 (38%)

S_5	289 (4.29)	0.0047	H→L+2 (93%)
S_6	273 (4.54)	0.0265	H-2→L+1 (72%)
S_7	270 (4.58)	0.0432	H→L+3 (62%)
S_8	270 (4.59)	0.0336	H-3→L (63%)
S_9	267 (4.56)	0.4484	H-1→L+1 (46%)
S_{10}	262 (4.73)	0.0133	H-1→L+2 (86%)
	***c*-BN-DAE**		
S_1	637 (1.95)	0.1805	H→L (99%)
S_2	414 (2.99)	0.0121	H→L+1 (97%)
S_3	404 (3.07)	0.0273	H-1→L (74%)
S_4	395 (3.14)	0.0087	H-2→L (63%)
S_5	371 (3.35)	0.1541	H→L+3 (50%)
S_6	347 (3.57)	0.3197	H→L+3 (46%), H→L+2 (34%)
S_7	339 (3.65)	0.0150	H-3→L (96%)
S_8	306 (4.06)	0.0816	H→L+4 (78%)
S_9	296 (4.19)	0.0851	H-4→L (42%), H→L+5 (35%)
S_{10}	295 (4.21)	0.0031	H-4→L (34%), H→L+5 (43%)
	***ap*-CC-DAE**		
S_1	383 (3.24)	0.0345	H→L (92%)
S_2	353 (3.51)	0.1267	H→L+1 (78%)
S_3	319 (3.89)	0.0073	H→L+2 (97%)
S_4	308 (4.02)	0.0715	H-1→L (53%)
S_5	298 (4.17)	0.0246	H→L+3 (91%)
S_6	295 (4.21)	0.0184	H-1→L+1 (76%)
S_7	286 (4.34)	0.3788	H-2→L (27%), H-2→L+1 (25%)
S_8	281 (4.41)	0.1233	H-2→L+1 (65%)
S_9	272 (4.56)	0.0202	H-3→L (77%)
S_{10}	265 (4.67)	0.0084	H-1→L+2 (81%)
	***c*-CC-DAE**		
S_1	623 (1.99)	0.0760	H→L (99%)
S_2	461 (2.69)	0.1547	H-1→L (93%)
S_3	427 (2.90)	0.0082	H→L+1 (97%)
S_4	391 (3.17)	0.0050	H-2→L (64%)
S_5	367 (3.38)	0.1712	H→L+3 (36%), H→L+2 (30%)
S_6	358 (3.47)	0.0054	H-3→L (86%)
S_7	345 (3.60)	0.2675	H→L+3 (52%)
S_8	325 (2.82)	0.1662	H-1→L+1 (75%)
S_9	314 (3.95)	0.0080	H-1→L+2 (89%)
S_{10}	301 (4.12)	0.1017	H-4→L (46%), H→L+4(38%)

Cartesian coordinates of ***ap*-BN-DAE**

N	1.72328000	0.63431800	-0.02280400
N	3.43559100	2.17449900	0.02736800
B	2.01145900	2.05601900	0.07326300
C	0.85689000	3.03773300	0.15780500
C	-0.45149800	2.47449700	0.12828900
C	-1.56475800	3.34202700	0.21106400
H	-2.56454200	2.92380500	0.20585600
C	-1.39005500	4.71291000	0.30379400
H	-2.25979800	5.36065800	0.36556000
C	-0.10188100	5.27083900	0.32372000

H	0.02571600	6.34649700	0.39749400
C	1.00087800	4.43462200	0.25376000
C	-0.63753600	1.02854800	0.04945500
C	0.41861400	0.15046600	-0.00966400
H	1.99966700	4.86439900	0.27514800
H	4.01804800	2.99464000	0.05408600
C	4.60131600	-1.76928900	-0.48898400
C	3.25792800	-1.40495600	-0.36666300
C	2.94783200	-0.05834000	-0.16967500
C	3.98778400	0.90705300	-0.12520800
C	5.32392100	0.53428800	-0.24429500
C	5.62276100	-0.81679600	-0.42401800
H	4.84900500	-2.81491000	-0.64071800
H	2.48632300	-2.15676400	-0.42737400
H	6.10958400	1.28293700	-0.20521300
H	6.65840200	-1.12704600	-0.52067700
C	-2.02520900	0.49048400	0.04367500
C	-2.89230500	0.56259700	-1.10301000
C	-2.62818700	-0.11224600	1.11936100
C	-4.12250200	0.00164200	-0.91429300
H	-2.59132500	1.02215700	-2.03797500
S	-4.25170600	-0.62925500	0.71498100
C	0.20845800	-1.31636000	-0.00413900
C	0.47082700	-2.13600000	1.14857000
C	-0.24780100	-2.03562100	-1.07918100
C	0.20732400	-3.46125900	0.95838100
H	0.85429400	-1.73711700	2.08051100
S	-0.37243100	-3.73298300	-0.67340800
C	-5.25656300	-0.11027200	-1.88506200
H	-4.97220300	0.34581200	-2.83722500
H	-5.52700500	-1.15428500	-2.07923600
H	-6.15700000	0.39908000	-1.52375800
C	-2.06819900	-0.35574100	2.48464000
H	-1.14635300	0.21823300	2.60482600
H	-2.76768100	-0.04977200	3.26933100
H	-1.82620900	-1.41261400	2.63898800
C	-0.61501000	-1.54374600	-2.44262100
H	-0.13441800	-2.13547600	-3.22832600
H	-1.69763800	-1.57852000	-2.60124000
H	-0.29764000	-0.50437100	-2.55159300
C	0.35701500	-4.59078000	1.92901600
H	0.74011100	-4.20998000	2.87940000
H	-0.59869000	-5.08919900	2.12541200
H	1.05587700	-5.35195600	1.56499500

Cartesian coordinates of ***c*-BN-DAE**

N	-1.84951200	0.40729600	-0.09207800
N	-3.70900700	1.76414300	-0.07282500
B	-2.29452300	1.77012300	-0.25213400
C	-1.27172000	2.88897500	-0.42561100
C	0.09685800	2.53856800	-0.26658800
C	1.05206900	3.54300600	-0.53329900
H	2.10361000	3.29847600	-0.56524500
C	0.67500300	4.84709600	-0.83238500
H	1.44399800	5.58826400	-1.02984000
C	-0.67379700	5.20000900	-0.91361300
H	-0.96613100	6.21856400	-1.14857000
C	-1.63048500	4.20898600	-0.73160300
C	0.49961000	1.13341000	-0.00252000
C	-0.49229100	0.03826300	-0.15584400
H	-2.68338600	4.45667600	-0.84345800
H	-4.35946700	2.53199600	-0.05328300
C	-4.32018300	-2.08633300	1.31507800
C	-3.06272800	-1.63569500	0.89864300
C	-2.95391300	-0.35460200	0.35723100
C	-4.09755200	0.48607200	0.32519100
C	-5.34807500	0.02793000	0.72272400
C	-5.45339900	-1.27729300	1.21078700
H	-4.40744300	-3.08493900	1.73108500
H	-2.19706400	-2.27110400	1.01221000
H	-6.21311500	0.68312200	0.68061900
H	-6.41977800	-1.65326100	1.53104100
C	1.81307100	0.77184100	0.23273400
C	2.89359800	1.52834000	0.79287100
C	2.30502100	-0.61324500	-0.23239700
C	4.04512600	0.84063600	1.02278300
H	2.78545000	2.54699600	1.14034000
S	3.98682100	-0.85258300	0.53887500
C	-0.08997900	-1.26018500	-0.33546000
C	-0.79791500	-2.36774100	-0.91447400
C	1.29535500	-1.68079300	0.17301400
C	-0.06449300	-3.48898700	-1.12089000
H	-1.82423800	-2.29740200	-1.25043500
S	1.62607300	-3.35174100	-0.59629700
C	5.28825800	1.37358100	1.65652800
H	5.14648400	2.41186400	1.96629300
H	5.56856600	0.78292400	2.53640300
H	6.13383900	1.33331500	0.95995400

C	-0.52375400	-4.76248600	-1.75101300
H	-1.56677600	-4.67697800	-2.06596600
H	0.08400300	-5.01489500	-2.62824500
H	-0.44260100	-5.60325200	-1.05185200
C	1.18943300	-1.87447600	1.70057400
H	0.38130200	-2.57609000	1.91575400
H	2.11596000	-2.26669500	2.12137200
H	0.95885300	-0.91790300	2.17859800
C	2.50646000	-0.50284200	-1.76139100
H	3.16984600	0.33711400	-1.97662000
H	2.94341700	-1.41532700	-2.16703600
H	1.54316100	-0.32519500	-2.24579000

Cartesian coordinates of ***ap*-CC-DAE**

N	1.71038900	0.65217200	-0.05630500
C	0.87189100	2.95300000	0.17412800
C	-0.45090100	2.44401200	0.12514400
C	-1.52876400	3.34674200	0.21981700
H	-2.54017500	2.95807300	0.19945300
C	-1.30745800	4.70942600	0.34364300
H	-2.15088900	5.38917700	0.41378200
C	0.00255600	5.20888900	0.38511400
H	0.17655500	6.27573400	0.48412600
C	1.07751300	4.33858300	0.30388100
C	-0.65760200	1.00628400	0.03100800
C	0.40743100	0.14906600	-0.03468200
H	2.09110300	4.72331200	0.34068100
H	3.84283000	3.18674900	0.12712800
C	4.57733300	-1.71476400	-0.59324900
C	3.23873200	-1.36485600	-0.46683200
C	2.93262200	-0.01959100	-0.21559300
C	3.96146700	0.96457900	-0.13089600
C	5.30743100	0.57560100	-0.25842700
C	5.60533000	-0.75787900	-0.48288200
H	4.83083600	-2.75206600	-0.78802900
H	2.47213800	-2.11642800	-0.57043700
H	6.09507800	1.31981700	-0.18977600
H	6.63981300	-1.07108400	-0.58584000
C	-2.04564600	0.47709600	0.03372200
C	-2.93429400	0.60547000	-1.09143600
C	-2.63268300	-0.16155300	1.09774700
C	-4.16702500	0.05115600	-0.89869900
H	-2.64611900	1.09622400	-2.01453500
S	-4.26941900	-0.64298600	0.70636100

C	0.23593500	-1.31945500	-0.01056900
C	0.57187100	-2.12120100	1.13602200
C	-0.26863100	-2.05617600	-1.05206800
C	0.31751300	-3.45146400	0.97381200
H	1.00098200	-1.70650000	2.04068300
S	-0.34592600	-3.74935200	-0.62167800
C	-5.32213400	-0.00978200	-1.84900000
H	-5.05223400	0.48037800	-2.78826900
H	-5.60884900	-1.04222300	-2.07812000
H	-6.20850200	0.49482200	-1.44862000
C	-2.04997500	-0.46353900	2.44174200
H	-1.13494100	0.11714600	2.57998800
H	-2.74380700	-0.20758200	3.24874900
H	-1.78935400	-1.52250800	2.53982800
C	-0.71850500	-1.58584100	-2.39819000
H	-0.30750100	-2.20737300	-3.19981500
H	-1.81012000	-1.59745000	-2.47989200
H	-0.38665000	-0.55723800	-2.55565400
C	0.53949400	-4.56876500	1.94465600
H	0.96219300	-4.17150800	2.87111900
H	-0.39419100	-5.08530900	2.19295700
H	1.23532700	-5.31793600	1.55129400
C	1.97877600	2.03538900	0.07856700
C	3.34093400	2.23390100	0.05158800

Cartesian coordinates of ***c*-CC-DAE**

N	-1.84498100	0.39926900	-0.02747300
C	-1.35593000	2.77189500	-0.43514700
C	0.02514400	2.52116000	-0.22618400
C	0.91485900	3.58710700	-0.47487000
H	1.98000700	3.41244300	-0.44911200
C	0.46426600	4.85284200	-0.82265200
H	1.18446100	5.64452000	-1.00435000
C	-0.90389900	5.09683600	-0.97569400
H	-1.26152300	6.08207800	-1.25679700
C	-1.79873300	4.05305600	-0.80097800
C	0.48938200	1.14786000	0.04757200
C	-0.48296200	0.04876700	-0.12291400
H	-2.85919800	4.21545500	-0.96097200
H	-4.30062500	2.61396600	-0.13264900
C	-4.22245200	-2.15640900	1.35697800
C	-2.98115700	-1.67001100	0.95753300
C	-2.92570900	-0.38569400	0.40421600
C	-4.09184500	0.43041300	0.33641100

C	-5.33355900	-0.09300400	0.72979000
C	-5.39282200	-1.38750200	1.22571100
H	-4.28142600	-3.15077800	1.78837600
H	-2.09312400	-2.26990600	1.09104600
H	-6.22635900	0.52190900	0.66839400
H	-6.34489800	-1.80408800	1.53931700
C	1.81102000	0.81104500	0.25918800
C	2.89131600	1.58404800	0.79539800
C	2.31490900	-0.56153300	-0.22802900
C	4.05396900	0.90883900	1.00716600
H	2.77854500	2.60215800	1.14307200
S	4.00312900	-0.78777900	0.53186200
C	-0.07604100	-1.24190200	-0.33332000
C	-0.77292000	-2.35072300	-0.92265700
C	1.31913600	-1.64949000	0.16287200
C	-0.02246900	-3.45658500	-1.15054400
H	-1.80448400	-2.29302400	-1.24420200
S	1.66830900	-3.30237500	-0.63564100
C	5.30324200	1.45596300	1.61604300
H	5.15676000	2.49350800	1.92596800
H	5.60573800	0.87049600	2.49197300
H	6.13588500	1.42228000	0.90382700
C	-0.46756200	-4.72753000	-1.79593000
H	-1.51342600	-4.65254500	-2.10370300
H	0.13895900	-4.95844200	-2.67970900
H	-0.36991100	-5.57613400	-1.10861600
C	1.22305900	-1.87080400	1.68716900
H	0.42730700	-2.58855000	1.89537600
H	2.15786200	-2.25619900	2.09576900
H	0.98175700	-0.92608500	2.18299900
C	2.50807200	-0.42641400	-1.75640200
H	3.15798900	0.42616200	-1.96239600
H	2.95633300	-1.32647600	-2.17711000
H	1.54034000	-0.25561500	-2.23465700
C	-3.67516900	1.73648600	-0.06581100
C	-2.31099700	1.70469400	-0.23475800

References

S1. Chan, J. C.-H.; Lam, W. H.; Yam, V. W.-W. A Highly Efficient Silole-Containing Dithienylethene with Excellent Thermal Stability and Fatigue Resistance: A Promising Candidate for Optical Memory Storage Materials. *J. Am. Chem. Soc.* **2014**, 136, 16994-16997.

S2 Ju, C. -W.; Li, B.; Li, L.; Yan, W.; Cui, C.; Ma, X.; Zhao, D. Modular Synthesis of Pentagonal and Hexagonal Ring-Fused NBN-Phenalenes Leading to an Excited-State Aromatization-Induced Structural Planarization Molecular Library. *J. Am. Chem. Soc.* **2021**,

143, 5903−5916.

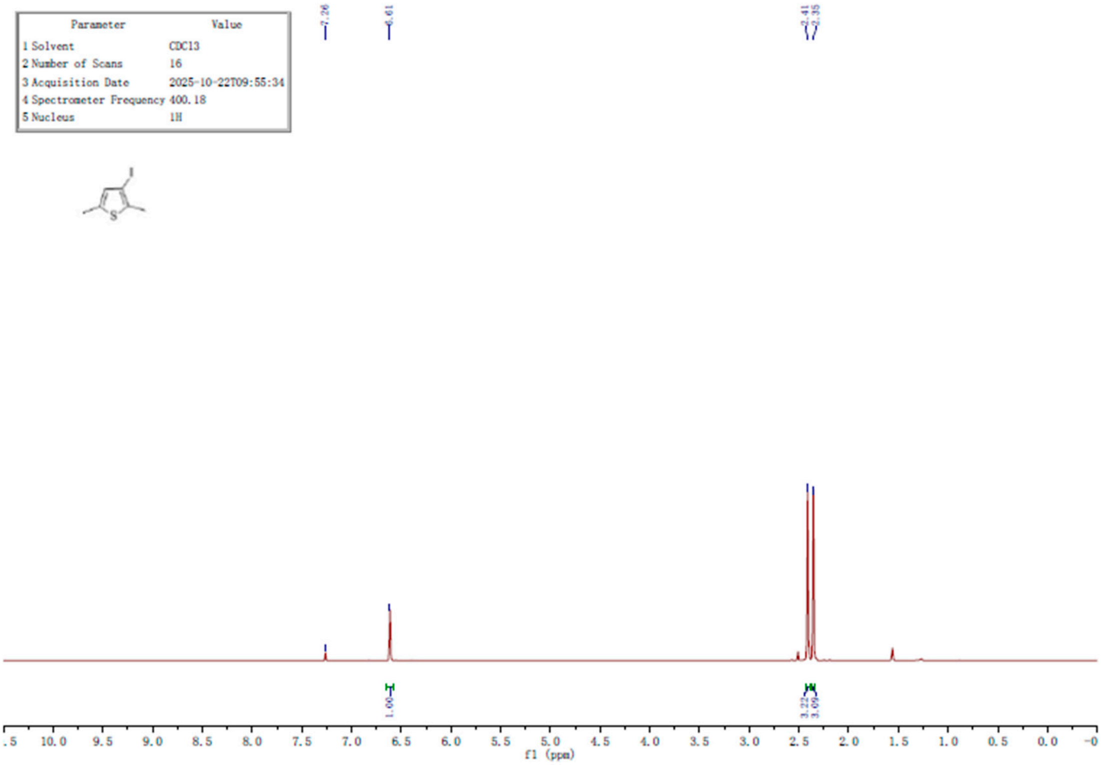

Figure S15. ^{1}H NMR of compound **2**

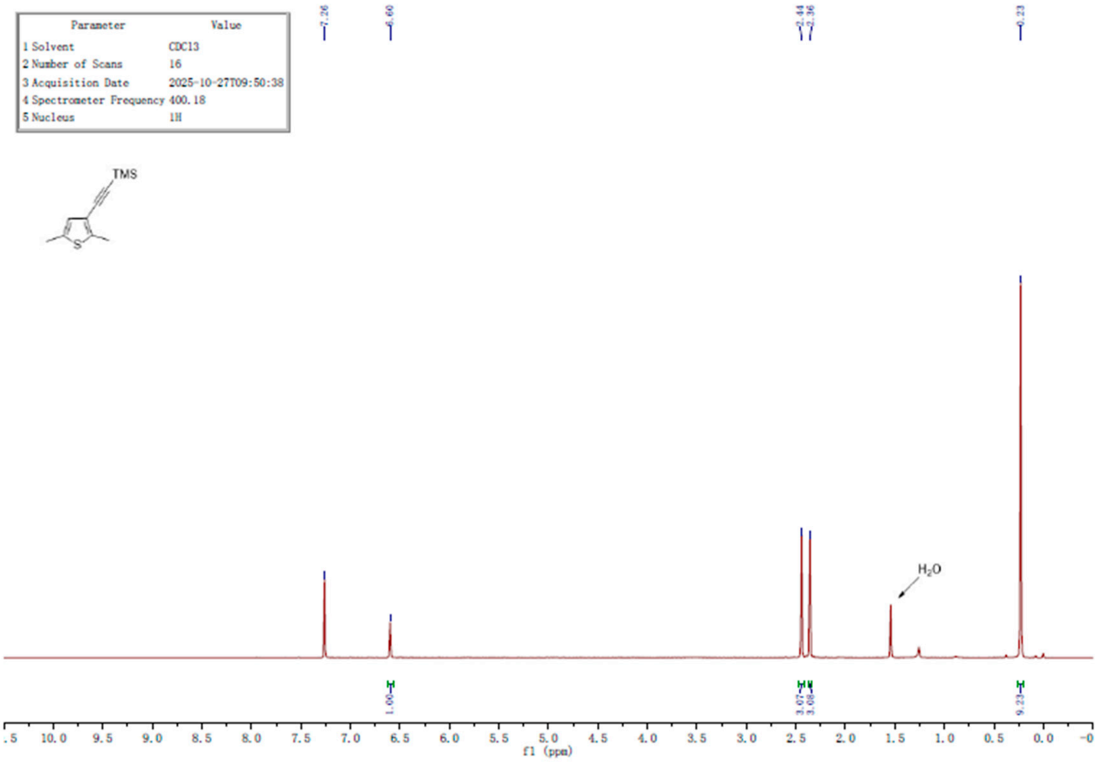

Figure S16. ^{1}H NMR of compound **3**

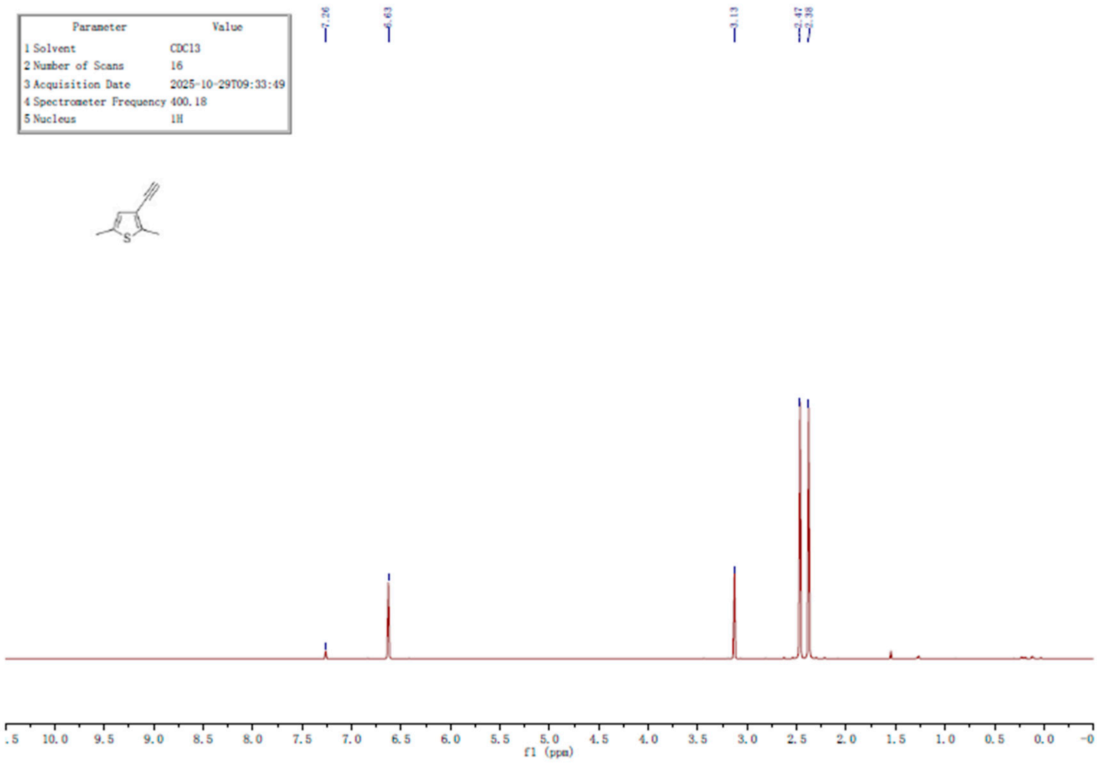

Figure S17. ^{1}H NMR of compound **4**

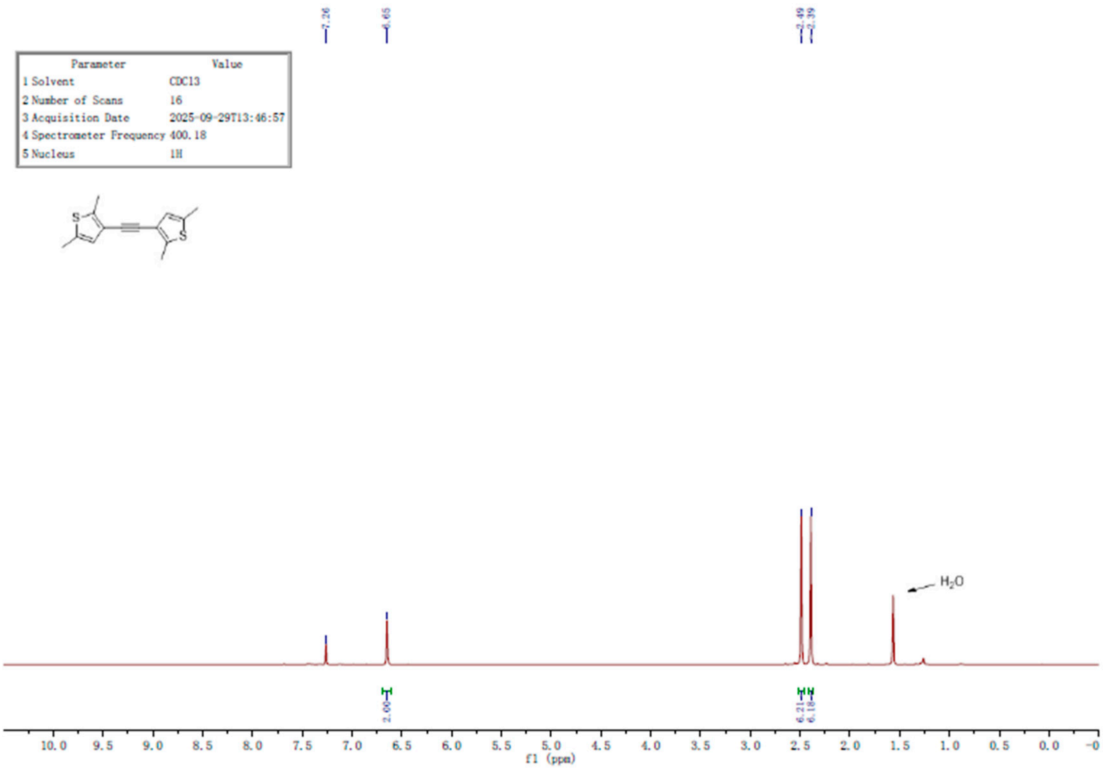

Figure S18. ^{1}H NMR of **BDTE**

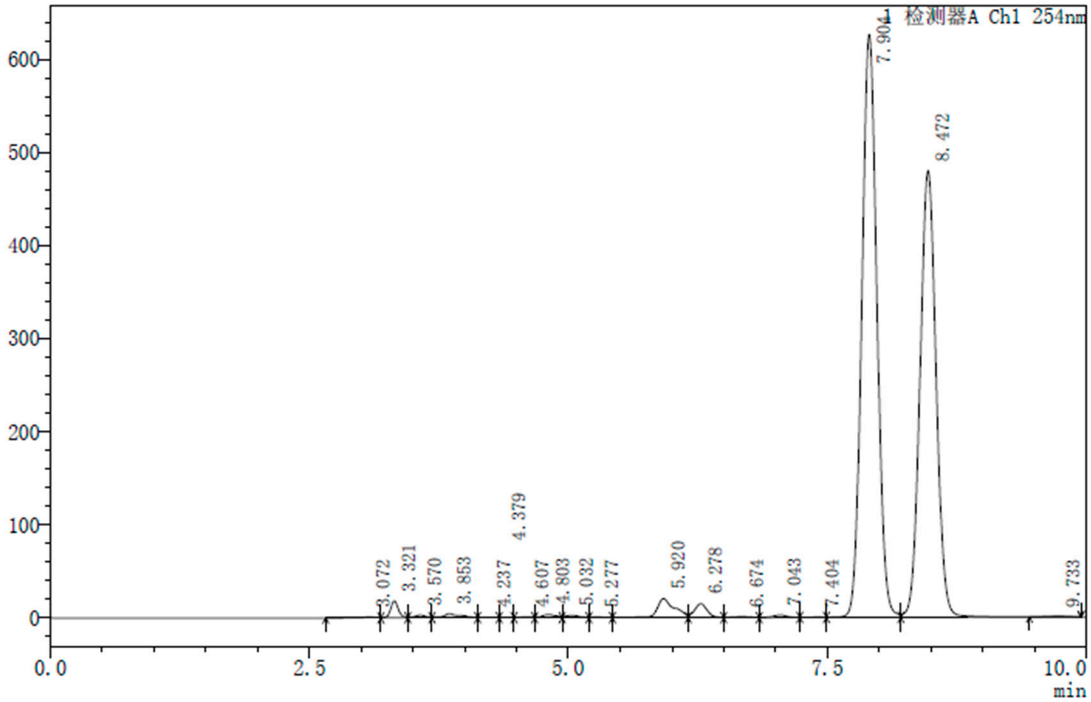

Figure S19. Analytic HPLC of **CC-DAE**

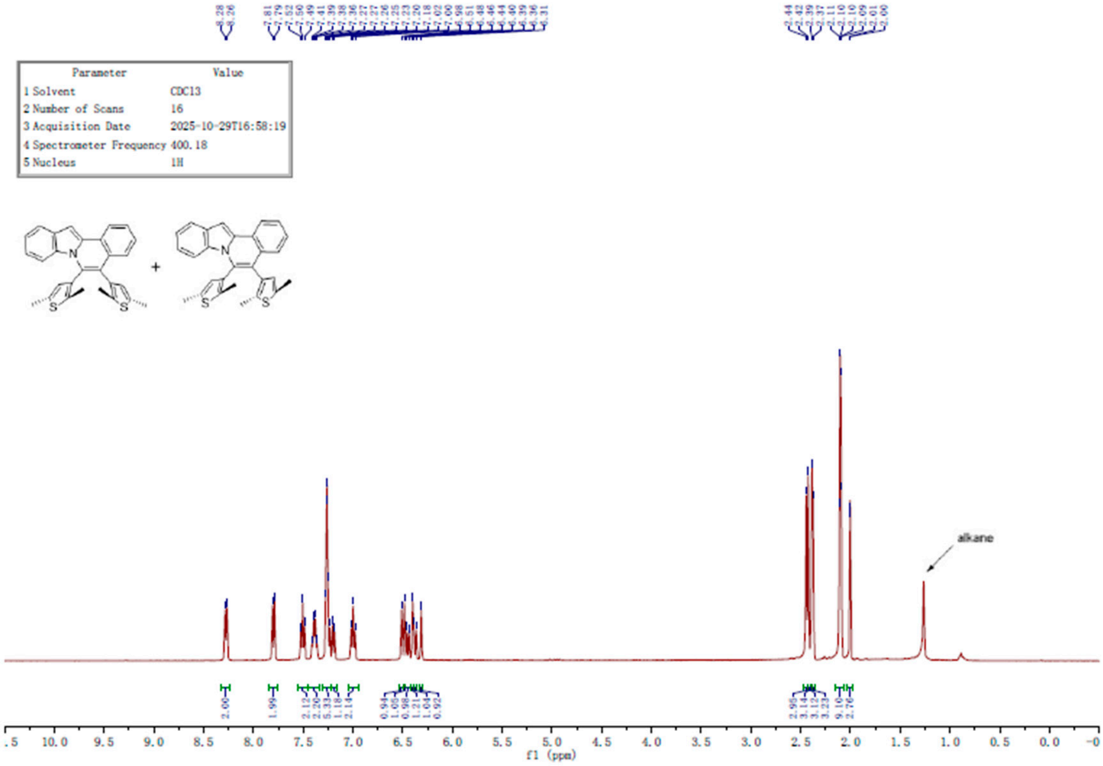

Figure S20. ^{1}H NMR of ***p*-CC-DAE** and ***ap*-CC-DAE** mixture

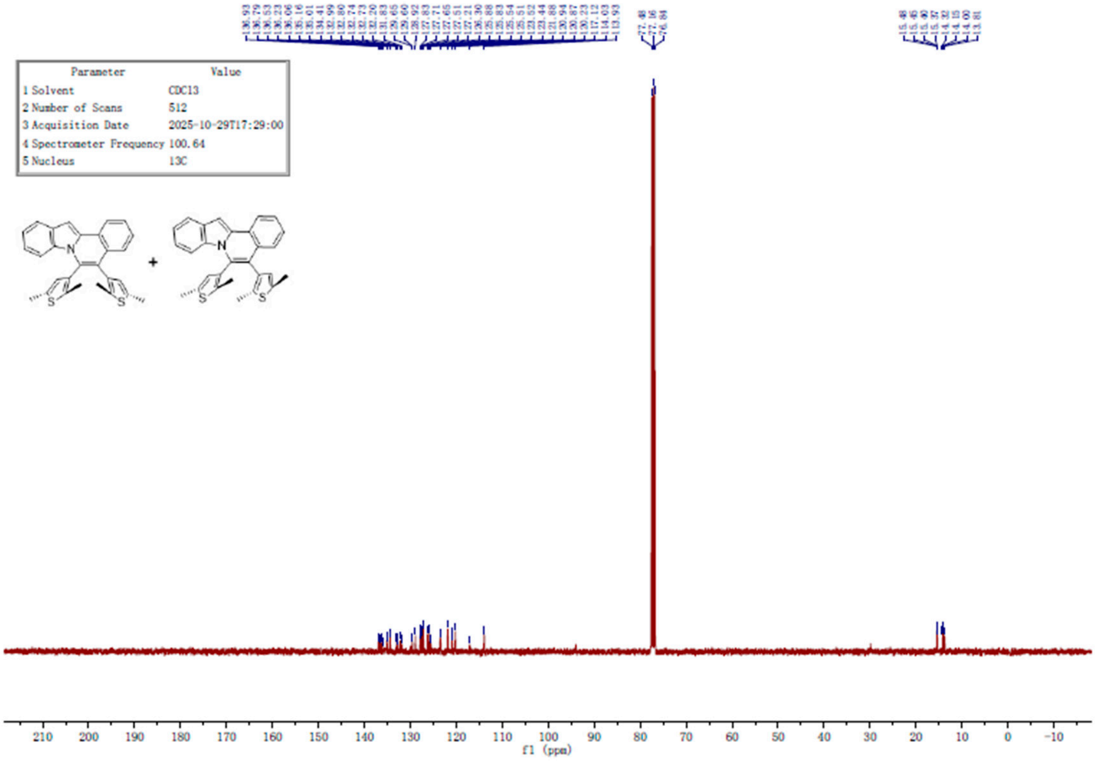

Figure S21. ^{13}C NMR of ***p*-CC-DAE** and ***ap*-CC-DAE** mixture

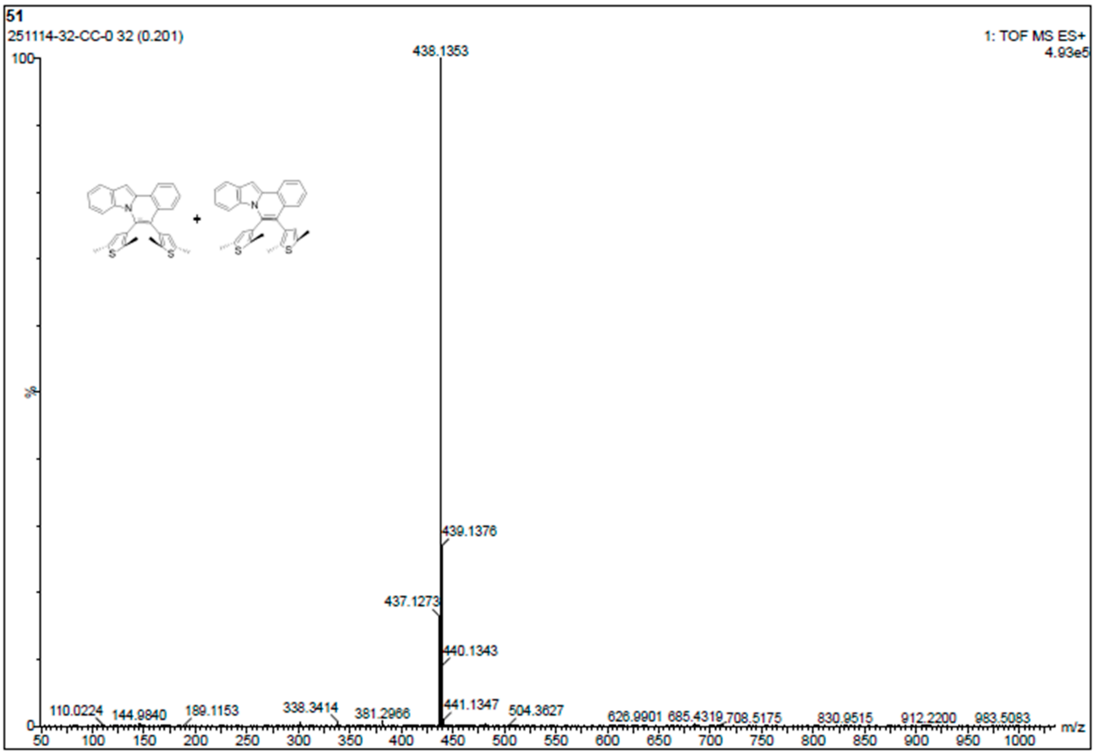

Figure S22. HRMS of ***p*-CC-DAE** and ***ap*-CC-DAE** mixture

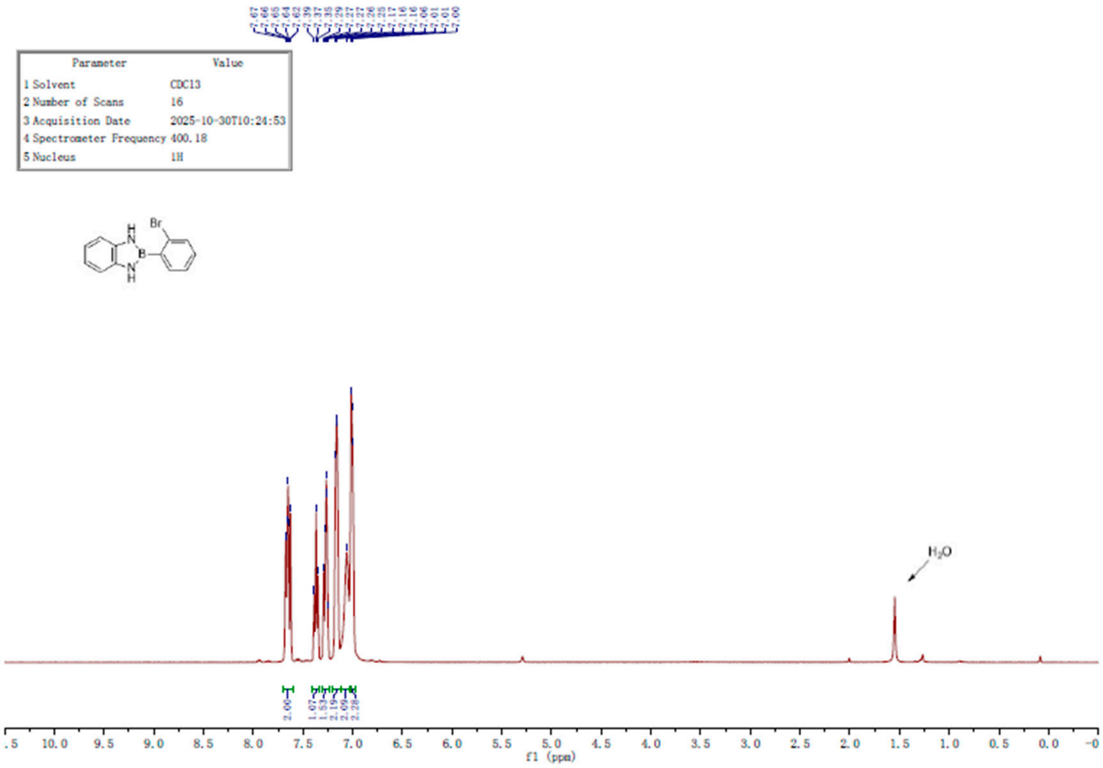

Figure S23. ^{1}H NMR of compound **8**

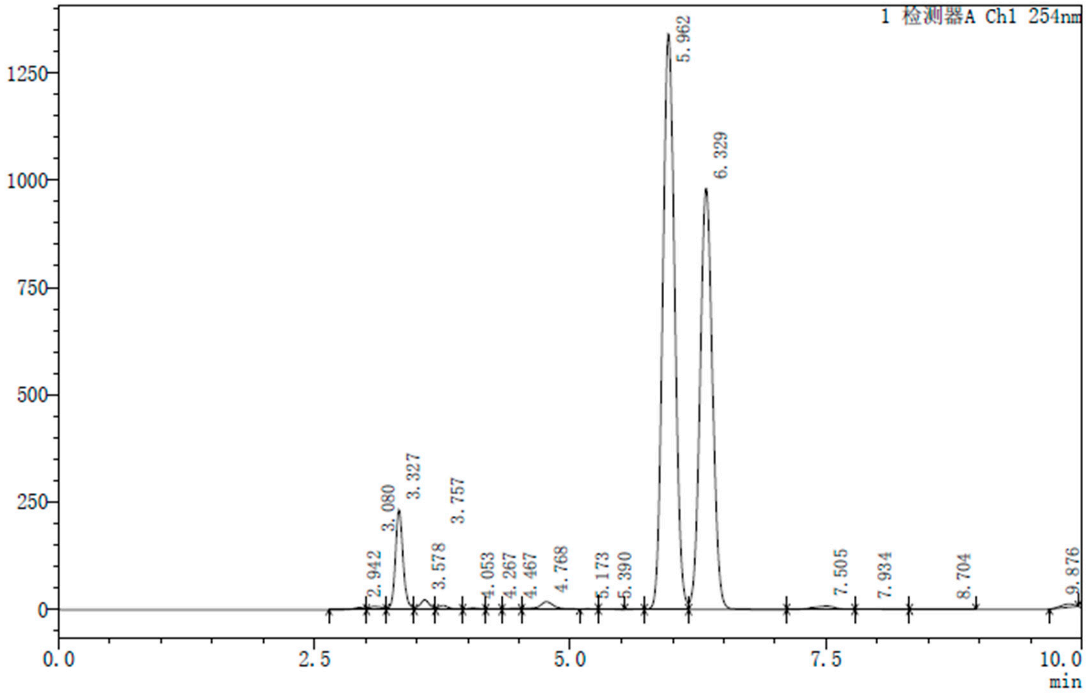

Figure S24. Analytic HPLC of **BN-DAE**

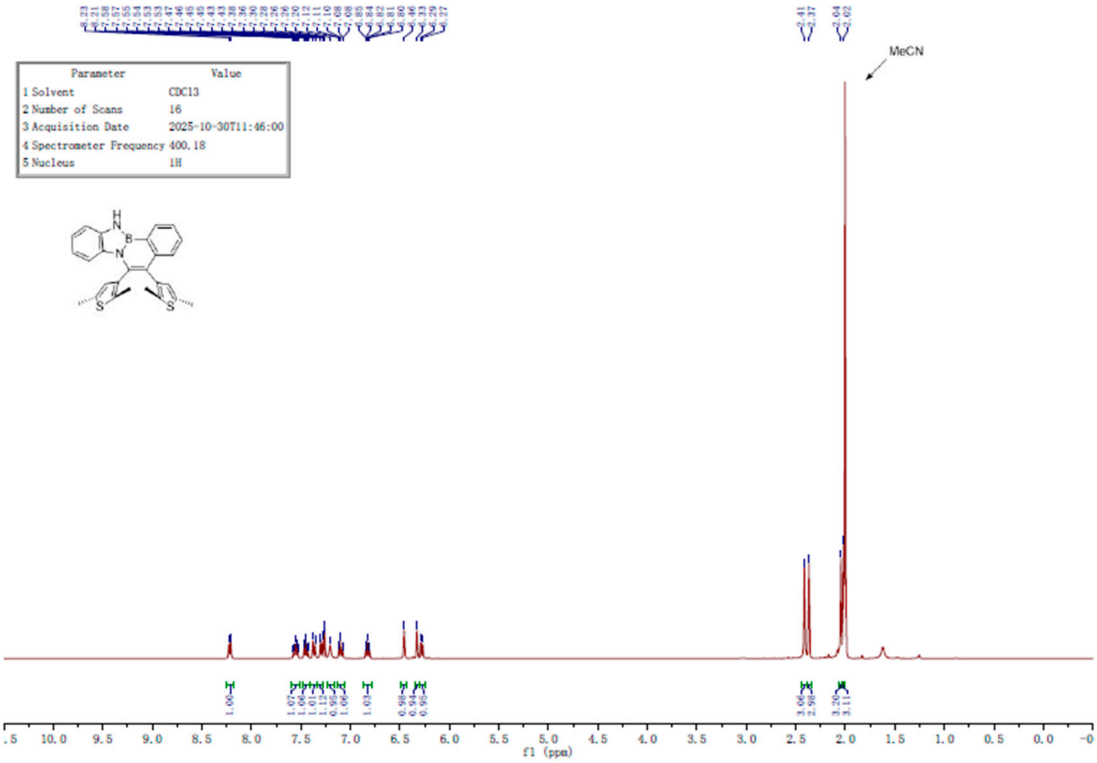

Figure S25. ^{1}H NMR of ***p*-BN-DAE** ($CDCl_3$)

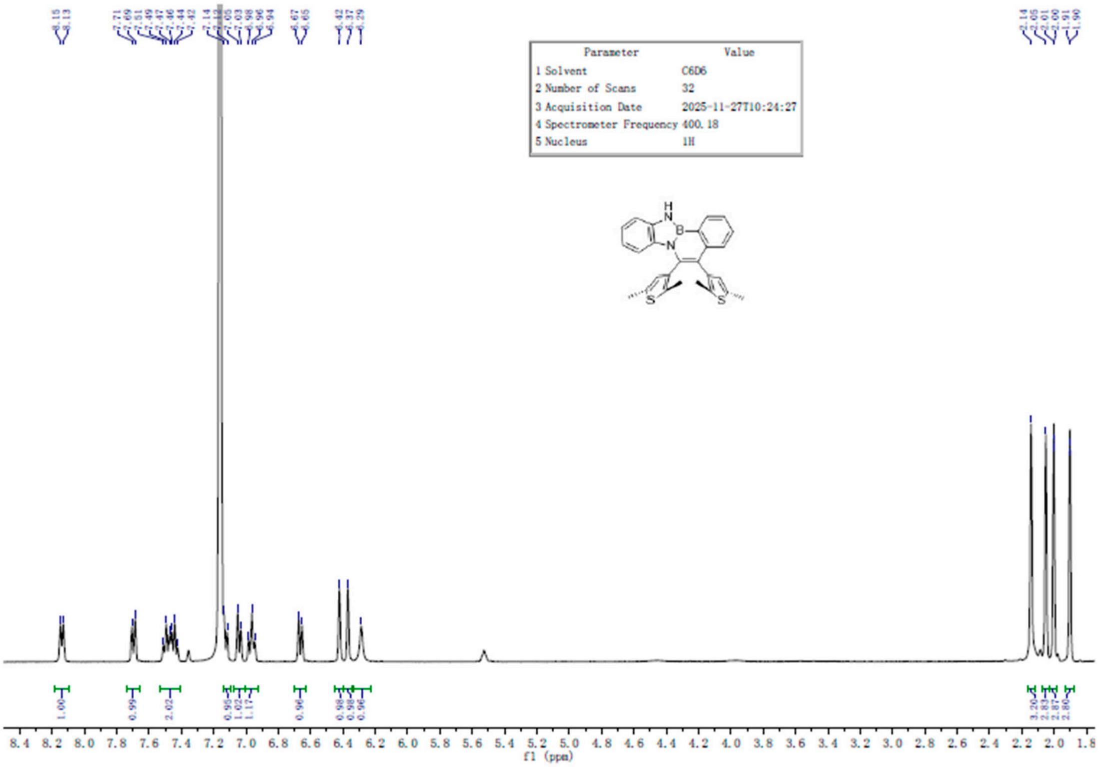

Figure S26. ^{1}H NMR of ***p*-BN-DAE** (C_6D_6)

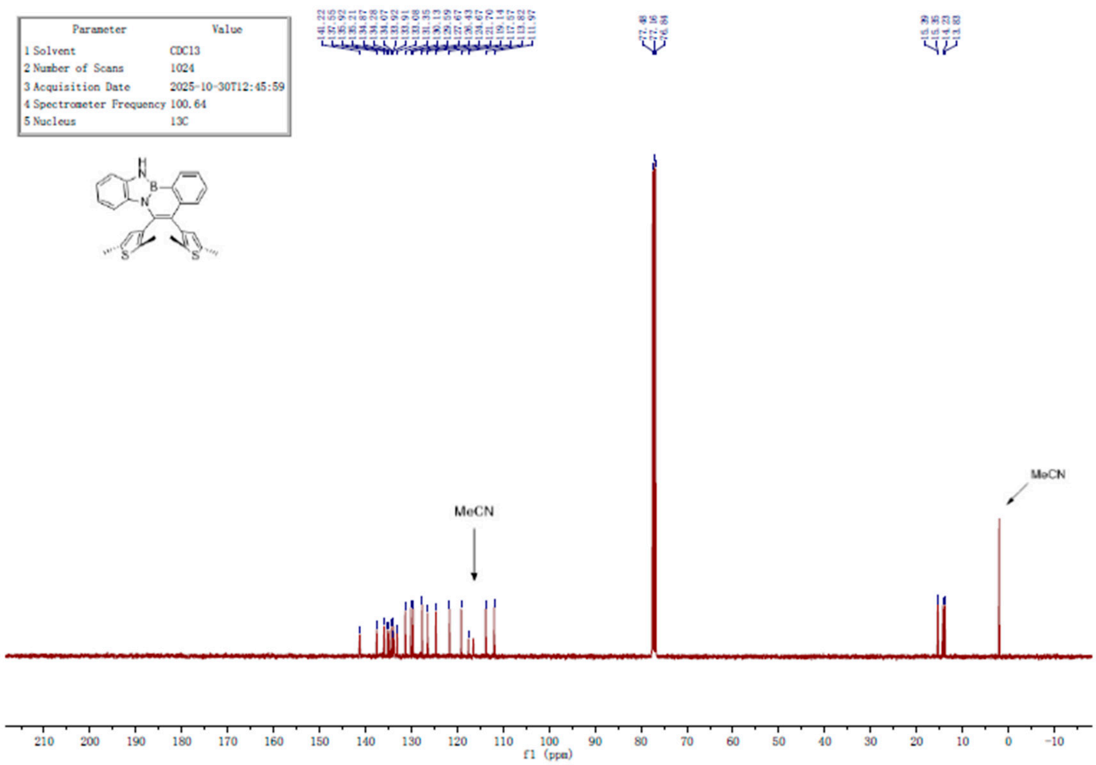

Figure S27. ^{13}C NMR of ***p*-BN-DAE**

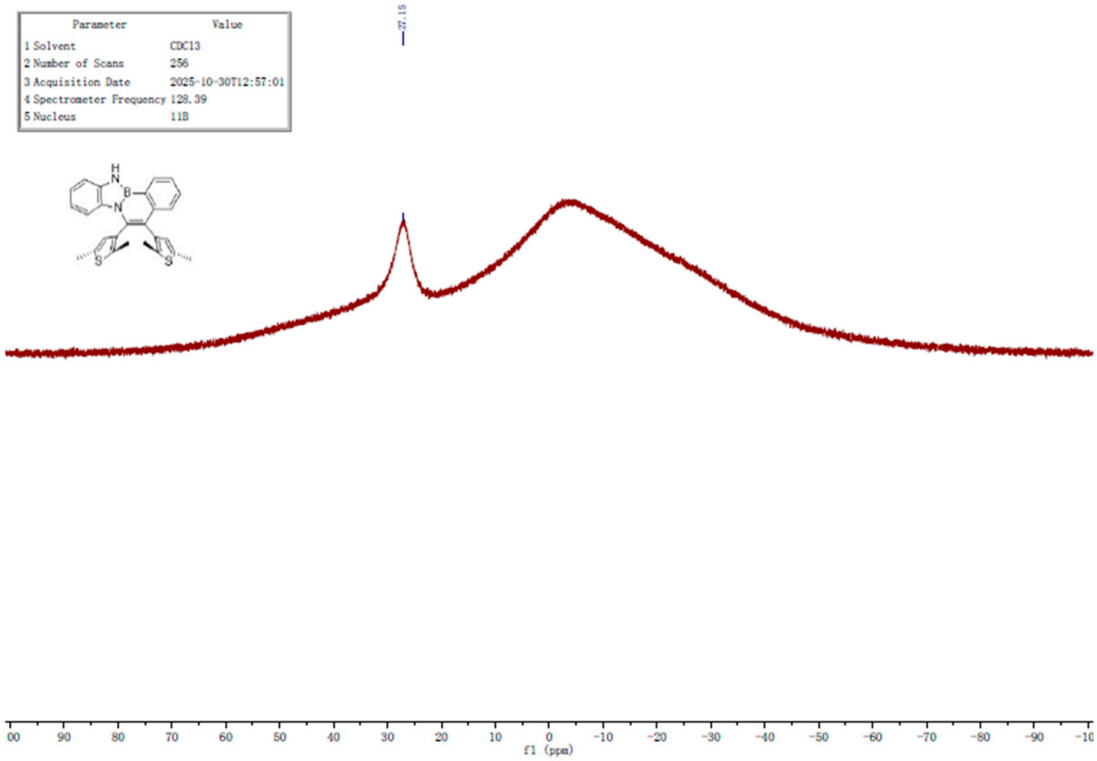

Figure S28. ^{11}B NMR of ***p*-BN-DAE**

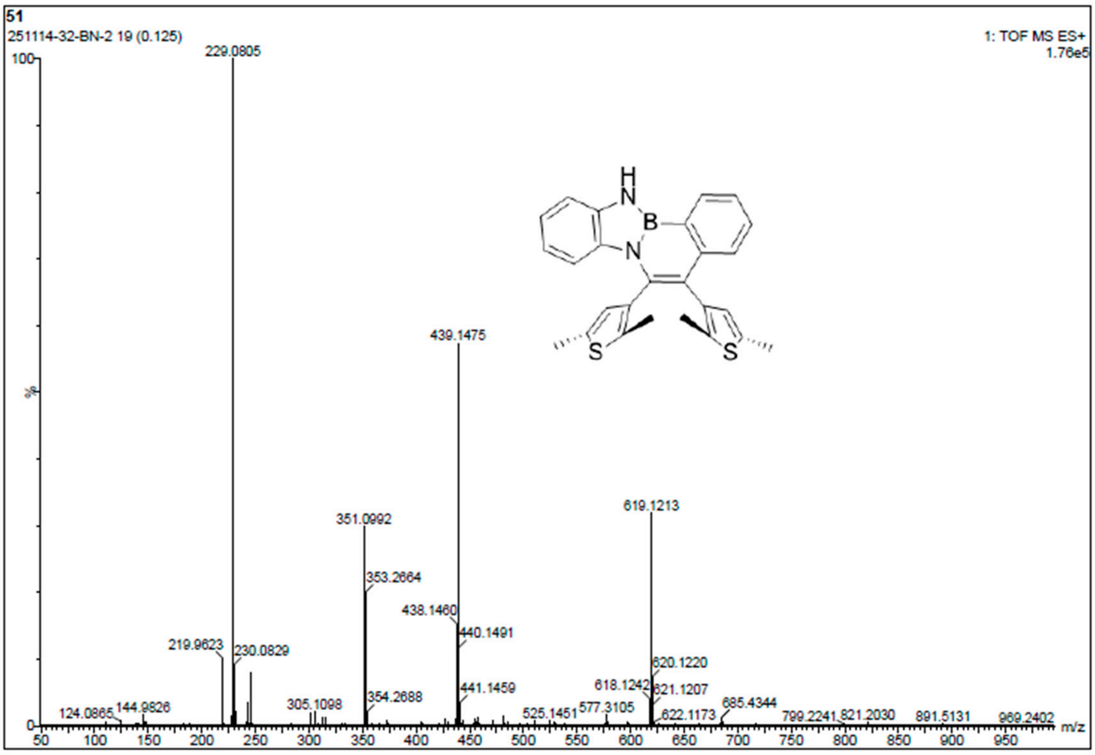

Figure S29. HRMS of ***p*-BN-DAE**

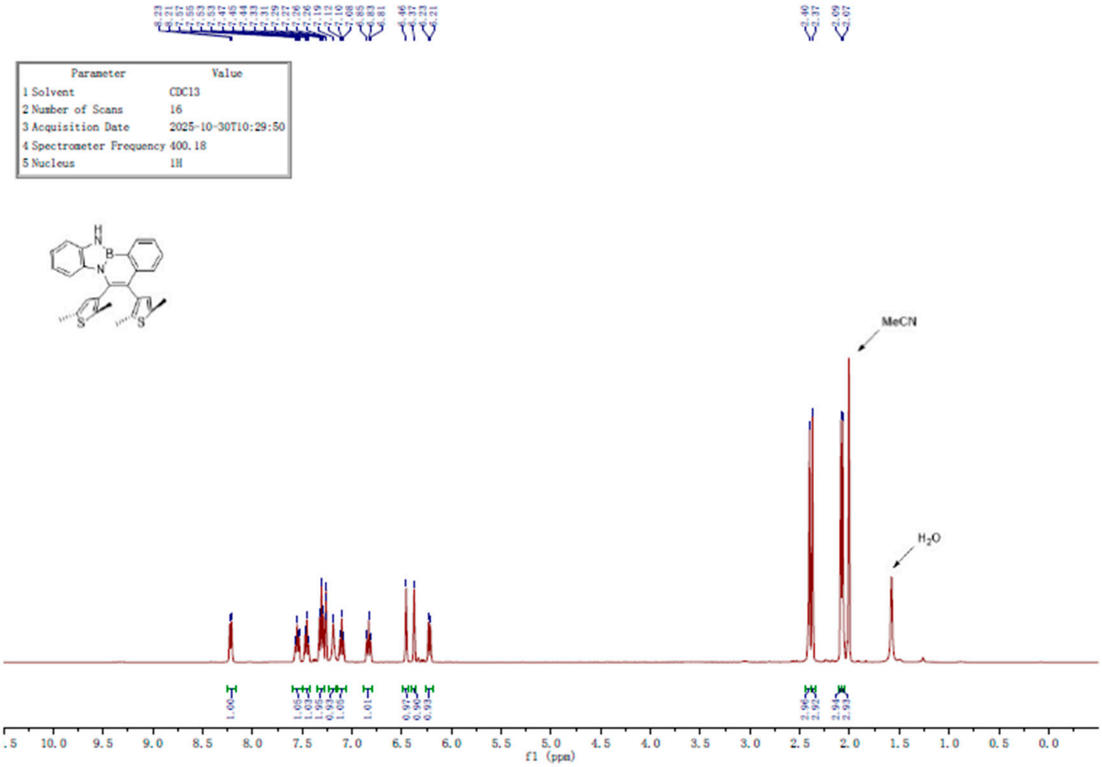

Figure S30. ^{1}H NMR of ***ap*-BN-DAE** ($CDCl_3$)

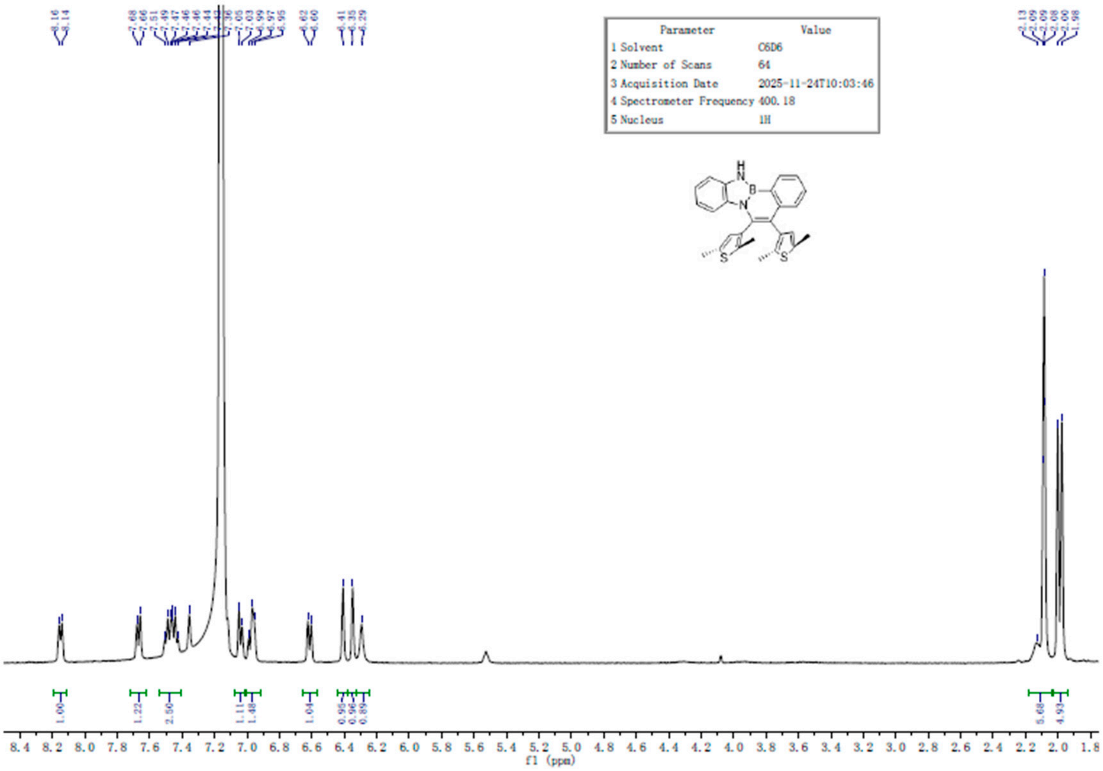

Figure S31. ^{1}H NMR of ***ap*-BN-DAE** (C_6D_6)

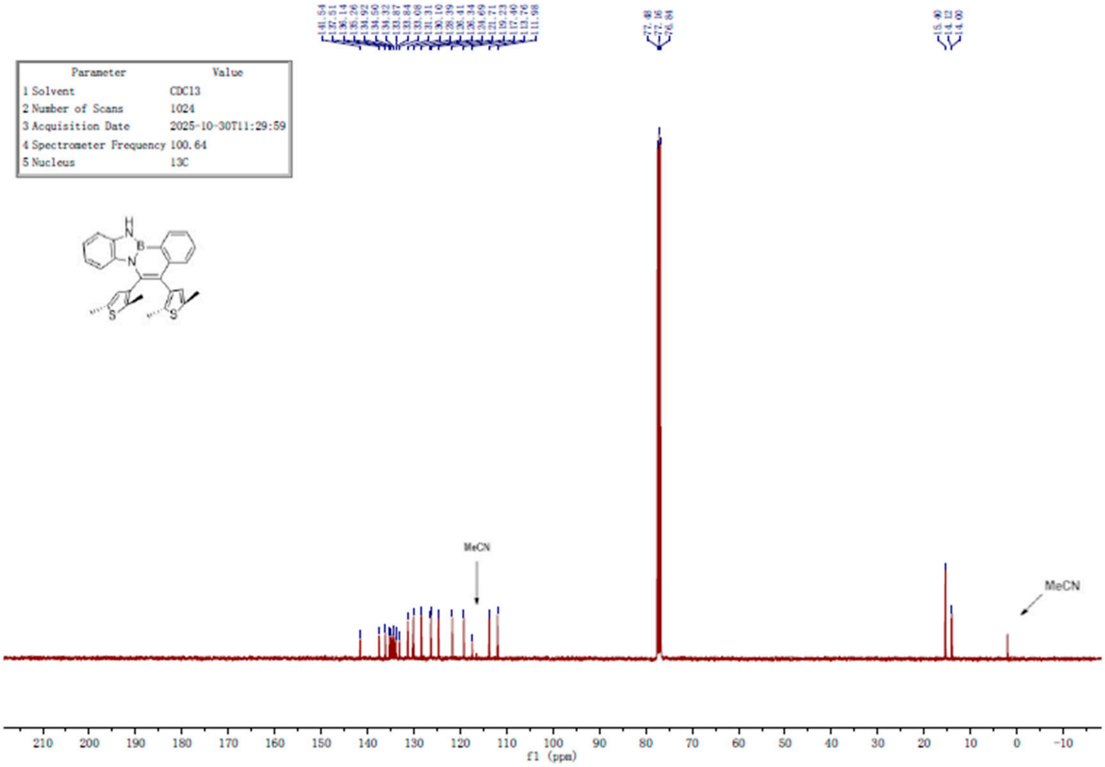

Figure S32. ^{13}C NMR of ***ap*-BN-DAE**

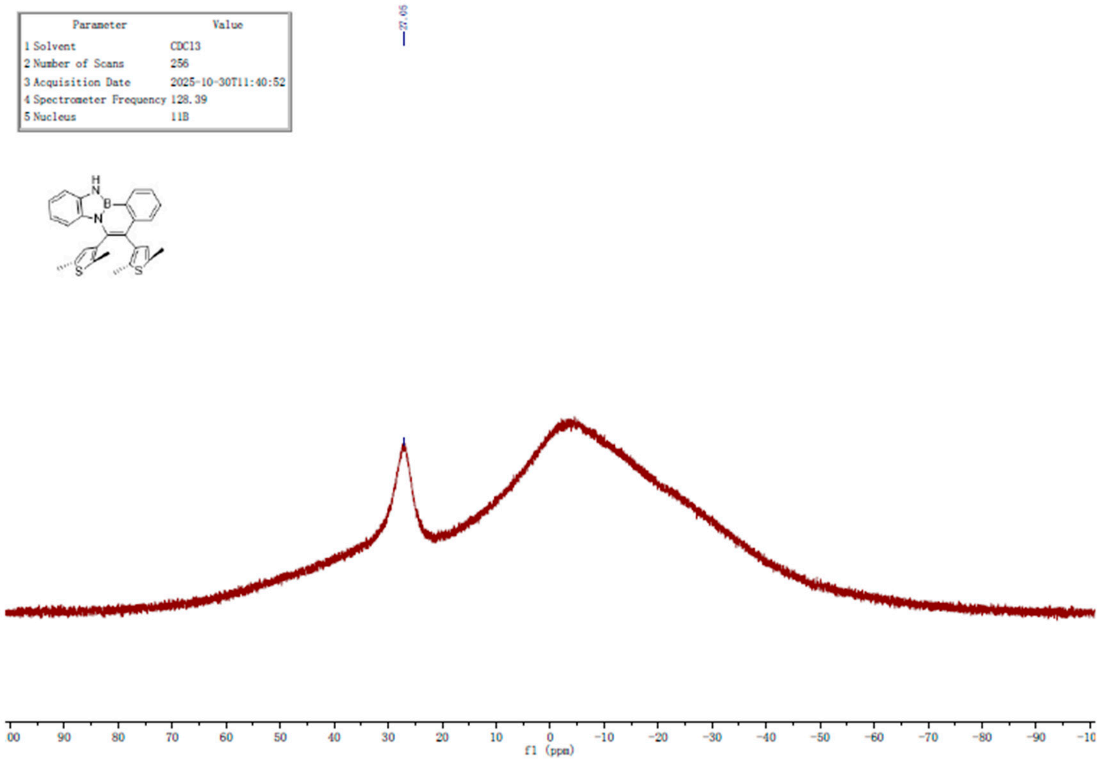

Figure S33. ^{11}B NMR of ***ap*-BN-DAE**

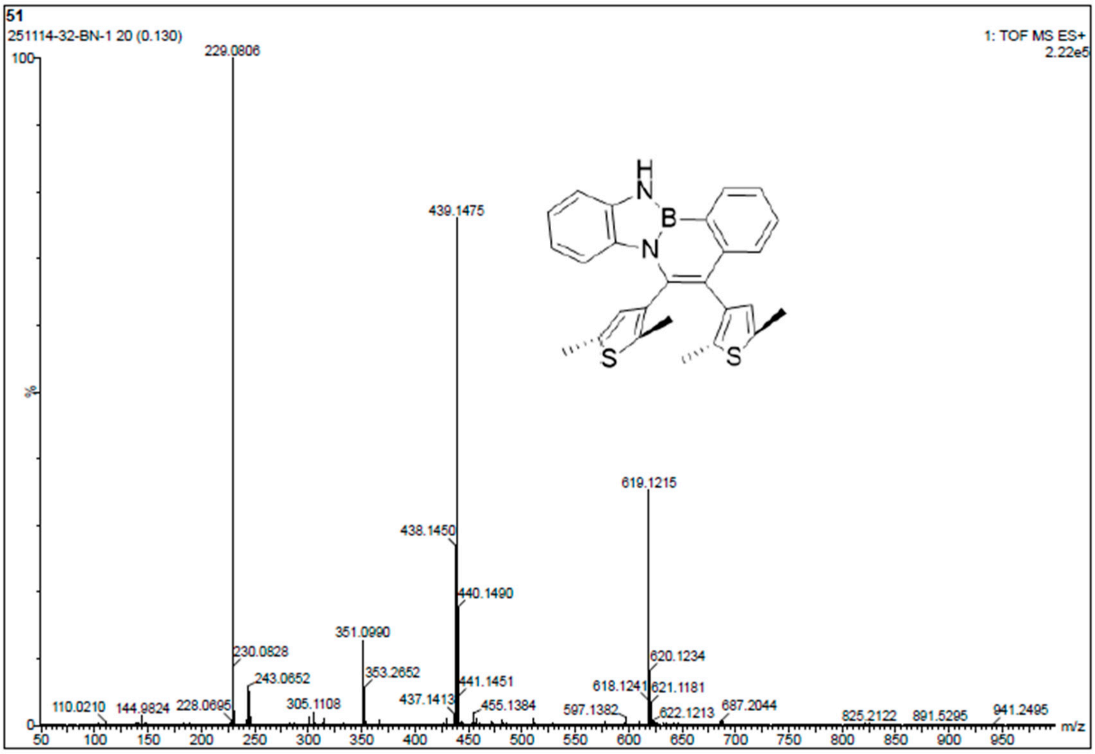

Figure S34. HRMS of ***ap*-BN-DAE**
